# GPS-Synchronized System for FPGA-Based Acceleration Measurement

**DOI:** 10.3390/s26216671

**Published:** 2026-10-22

**Authors:** Paulo Realpe-Muñoz, Jorge Erazo-Aux, Javier Cortes-Carvajal, John Erazo-Aux

**Affiliations:** 1 Faculty of Engineering, Institucion Universitaria Antonio José Camacho, Cali 760046, VA, Colombia; jherazo@admon.uniajc.edu.co (J.E.-A.); jcortes@admon.uniajc.edu.co (J.C.-C.); 2 SSI INGENIERIA S.A.S., Cali 760033, VA, Colombia; johnerazo@ssi.com.co

**Keywords:** Global Positioning System (GPS), synchronization, Pulse per Second (1PPS), Field-Programmable Gate Array (FPGA), Digital Phase Locked Loop (DPLL), accelerometer

## Abstract

This paper proposes an autonomous, resource-efficient, FPGA-based synchronization architecture for distributed accelerograph networks. Unlike existing FPGA implementations that focus primarily on acceleration acquisition, the proposed design integrates GPS-disciplined synchronization, synchronized acceleration acquisition, and real-time synchronization error estimation on a single FPGA platform, enabling autonomous sensing nodes without centralized clock distribution. The architecture was evaluated under various weather conditions, demonstrating reliable performance in real-world deployments. The architecture uses the Global Positioning System (GPS) one Pulse-Per-Second (1PPS) signal as the timing reference and integrates a Digital Phase-Locked Loop (DPLL), two frequency dividers, and a synchronization error estimation module. The DPLL generates a 1.024 MHz master clock, from which a 1 kHz sampling signal (Ext_Sync) and a synchronized 1 Hz signal (Ext_1Hz) are derived. The design was implemented in VHDL and Verilog, synthesized on a Cyclone V 5CSEBA6U23I7 FPGA, and experimentally validated on a DE10-Nano development board. The design was evaluated through hardware synthesis, synchronization accuracy under three weather conditions (cloudy, moderate rain, and clear sky), acceleration measurements, frequency-response characterization, and statistical analysis using analysis of variance (ANOVA). The proposed architecture achieved a mean synchronization error of 4.07 µs. Furthermore, the measured frequency response satisfied the requirements of the International Society of Explosives Engineers (ISEE) over approximately 40% of the specified frequency range, demonstrating the feasibility of the proposed architecture for synchronized accelerograph networks in scientific instrumentation and seismic monitoring applications.

## 1. Introduction

The GPS is a fundamental technology in modern applications, including navigation, location tracking, passive radar systems for air target detection [1], synchronization of telecommunications networks [2,3], meteorology, and measurement of structural displacement using accelerometers [4,5,6], among others. GPS provides precise and accurate timing references over large geographical areas through onboard atomic clocks, which exhibit high stability with timing fluctuations of less than a few tenths of a nanosecond [7].

This timing accuracy enables the synchronization of clocks between different devices and systems, supporting operations that require accurate temporal coordination and real-time data analysis [8,9]. Other timestamping technologies rely on standards and protocols such as the IEEE 1588-2019 Precision Time Protocol (PTP) [10] and the Network Time Protocol (NTP) [11] to meet synchronization requirements. However, network-induced delays, protocol overhead, and processing latency can degrade synchronization performance and limit timing accuracy.

A common approach for generating stable and uniform timing references in GPS-based synchronization systems is to use Phase-Locked Loops (PLLs) [12]. PLLs are feedback control systems widely used in telecommunications, electronics, and control engineering to synchronize the phase of an output signal with a reference signal, enabling clock generation in digital circuits, frequency stabilization in communication systems, and signal demodulation in radio receivers. Although conventional PLLs have been widely used for synchronization applications, DPLLs have gained significant attention due to their flexibility, programmability, and suitability for FPGA implementation [13].

In seismic instrumentation, accelerographs equipped with Micro-Electro-Mechanical Systems (MEMSs) accelerometers measure acceleration in three dimensions. These measurements provide indirect information on displacement, inclination, rotation, shock-wave vibrations, velocity, frequency content, and movement patterns [14,15]. This information is essential for earthquake detection, early warning systems, structural health monitoring, microtremor logging, and risk mitigation [16]. The effectiveness of these applications depends on high-quality and reliable synchronization, accurate time reference generation, and consistent data acquisition across geographically distributed measurement systems. Although synchronization techniques have been investigated for a wide range of devices and applications, FPGA-based synchronous sampling for accelerographs remains largely unexplored.

To the best of our knowledge, although previous studies have demonstrated FPGA-based acceleration acquisition and GPS-assisted timing, they focus on data acquisition and do not address end-to-end synchronization of the acceleration measurement process. Furthermore, existing implementations do not integrate autonomous synchronization for distributed sensing nodes and do not provide a real-time estimation of synchronization error in an FPGA. Therefore, the timing quality of each measurement remains unknown during operation, limiting the deployment of distributed accelerograph networks that require microsecond- or nanosecond-level synchronization.

To address these limitations, this paper proposes an autonomous FPGA-based synchronization architecture that integrates GPS-disciplined clock generation, synchronized acceleration sampling, and real-time synchronization error estimation into a single hardware platform. Unlike conventional approaches, each sensing node operates independently with its own GPS receiver and FPGA, eliminating the need for centralized clock distribution while maintaining synchronized acceleration sampling across distributed measurement stations. The proposed design achieves a mean synchronization error of 4.07 µs between the GPS 1PPS reference and a 1 Hz internal signal. However, temporal fluctuations in the synchronization error are observed during the operation.

The main contributions of this study are summarized as follows:

1. A novel autonomous FPGA-based synchronization architecture that synchronizes the entire acceleration measurement system, from the GPS 1PPS reference to the sensor sampling instant, on a single hardware platform.
2. A real-time synchronization error estimation that continuously monitors the temporal offset between the GPS reference and the internally generated synchronization signals.
3. An autonomous distributed synchronization approach in which each sensing node independently generates GPS-disciplined internal signals using its own GPS receiver and FPGA, thereby eliminating the need for centralized clock distribution.
4. Experimental validation using single-sensor, dual-sensor, and distributed dual-FPGA configurations, including synchronization performance under clear-sky, cloudy, and moderate-rain (2–15 mm/h of precipitation) conditions, supported by ANOVA-based statistical analysis.
5. Experimental characterization of the dynamic response of the proposed architecture across the 2–175 Hz frequency range demonstrates compliance with the ISEE amplitude-response requirements over approximately 40% of the evaluated frequency range.
6. An efficient FPGA implementation that requires only 1163 LUTs and 606 FFs (<1%) demonstrates the suitability of the proposed architecture for embedded accelerograph systems used in scientific instrumentation and distributed seismic monitoring.

The remainder of this paper is organized as follows: Section 2 reviews related works. Section 3 provides theoretical background on DPLL, synchronization and measurement of MEMS accelerometers. Section 4 describes the FPGA-based acceleration measurement and synchronization system. Section 5 details the experimental setup, followed by the experimental results in Section 6. Finally, conclusions and further work are presented in Section 7.

## 2. Related Works

Several FPGA-based implementations for accelerometer interfacing and synchronization-related applications have been reported in the literature. In [17], an I^2^C protocol interface implemented in Verilog code is presented, enabling data exchange between an FPGA and an MPU6050 accelerometer (InvenSense Inc., San José, CA, USA). Similarly, ref. [18] presents an FPGA-based monitoring system built on the Zynq-7000 platform with embedded Linux for remote real-time monitoring of devices connected to the Internet. The proposed system acquires data from triaxial accelerometers using the Serial Peripheral Interface (SPI).

In [19], an FPGA-based acceleration measurement system is implemented on the NI sbRIO-9632 platform. The system is developed in LabVIEW FPGA and synthesized in VHDL for hardware implementation. Experimental results show that the acceleration measurement module utilizes approximately 15% of the available FPGA resources while operating at 44 MHz. In [20], an FPGA-based data-logging system is proposed for low-power triaxial accelerometers, allowing operation independent of the sensor orientation. The proposed approach incorporates a vector rotation algorithm that reduces computational complexity.

In [21], a GPS-based synchronization approach is proposed for wireless sensor nodes, offering a more accurate and deterministic alternative to conventional wireless synchronization protocols. In [22], an FPGA-based parallel data concentrator is developed for seismographs to improve the efficiency of data transmission in seismic exploration. Similarly, ref. [23] presents a real-time seismic data processing model based on differential Global Navigation Satellite System (GNSS) techniques, improving positioning accuracy through single- and double-difference models.

In [24], a high-sensitivity digital accelerometer is integrated into the next-generation Wireless Smart Sensor (WSS) platform, XNode. The platform provides a hardware and software framework for embedded computational analysis, and its performance is validated by comparison with high-sensitivity wired accelerometers. In [25], a software-based synchronization method is proposed for wireless accelerometers, enabling synchronized sampling to accurately monitor system conditions.

Beyond accelerometer-specific applications, time, phase, and frequency synchronization are fundamental requirements in modern systems, including distributed radar networks, wireless sensor networks, fifth-generation (5G) communication systems, Software-Defined Radio (SDR), and Low Earth Orbit (LEO) satellite constellations. GNSS-based synchronization solutions, particularly GPS-Disciplined Oscillators (GPSDOs), are widely adopted for their high timing accuracy and cost-effectiveness. In [26], the accuracy of sub-10 ns synchronization is demonstrated for coherent radar applications using phase alignment and fast-locking PLLs to maintain system coherence.

Recent research has focused on improving the reliability and independence of GPS-disciplined synchronization systems using digital methods and new control strategies. In [27], an FPGA-based GPSDO with adaptive drift correction maintains timing deviations below 80 ns, even after extended outages of the GNSS signal. Additionally, ref. [28] proposes a two-stage control strategy that combines Sliding Mode Control (SMC), Model Predictive Control (MPC), and a Linear Quadratic Regulator (LQR) to accelerate convergence and enhance long-term synchronization stability.

In [29], integrating a cesium atomic clock enables precise synchronization when GNSS signals are not available, although it increases both cost and system complexity. Meanwhile, the compact digital servo clock proposed in [30] achieves excellent frequency stability and timing accuracy of ±10 ns by combining an Oven-Controlled Crystal Oscillator (OCXO) with GNSS references. In [31], a GNSS synchronization chip based on a Time-to-Digital Converter (TDC) is introduced, providing 1 ns timing resolution. In contrast ref. [32] presents a GNSS-independent synchronization technique for SDR systems using calibration methods.

Finally, the commercial seismic instrumentation market is led by manufacturers such as REF TEK Systems [33], Kinemetrics [34], Nanometrics [35], Güralp [36], and GeoSIG [37]. These manufacturers rely on modern technologies for digital data collection and high-precision GNSS-based time synchronization, and they support standard protocols such as SeedLink, ensuring interoperability among seismic monitoring networks. However, acquiring, deploying, and maintaining these commercial systems often entails high costs and dependence on proprietary technology.

The reviewed literature demonstrates significant advances in FPGA-based sensing platforms, wireless accelerometer systems, and GNSS-disciplined synchronization techniques. These studies have improved timing accuracy, synchronization robustness, and embedded data acquisition for a wide range of applications. However, most existing solutions are intended for timing, communication, or general sensing applications and do not integrate GPS-disciplined synchronization with real-time acceleration acquisition in a single FPGA-based platform. In addition, the combined implementation of timing synchronization, phase alignment, acceleration acquisition, and synchronization error estimation for distributed accelerograph systems has received little attention in the literature.

To our knowledge, no previous study has reported an FPGA-based GPS synchronization system specifically designed for real-time acceleration measurements in accelerographs. The proposed architecture integrates GPS-based timing synchronization, phase alignment, acceleration data acquisition, and synchronization error estimation on a single FPGA platform. This design reduces hardware complexity, communication delays, and system cost while preserving the flexibility and scalability of FPGA-based designs.

## 3. Theoretical Background

This section introduces the theoretical background on Digital Phase-Locked Loops (DPLLs), MEMS accelerometers, and the synchronization procedure used for acceleration measurements with the ADXL355 sensor (Analog Devices Inc., Wilmington, MA, USA).

### 3.1. DPLL

A DPLL is a digital feedback control system that generates an output signal whose phase is synchronized with a reference input signal. Because phase synchronization inherently requires frequency synchronization, a DPLL first achieves frequency lock and can subsequently track the reference frequency accurately or generate an output frequency that is an integer multiple of it. These characteristics make DPLLs well-suited for applications such as digital clock synchronization, frequency synthesis, signal demodulation, and robust signal recovery in noisy communication channels [38].

DPLLs are widely used in telecommunications, computing, and other digital electronic systems because they provide accurate frequency and phase synchronization, including the generation and distribution of synchronized clock signals in digital systems such as microprocessors [38]. Analog and digital PLLs are based on the same feedback principle and comprise four main functional blocks: a Phase Detector (PD), a low-pass loop filter, a Voltage-Controlled Oscillator (VCO) and a frequency divider [39]. In digital implementations, the VCO used in conventional analog PLLs is replaced by a Numerically Controlled Oscillator (NCO). This substitution enables an efficient implementation on FPGAs.

The PD measures the phase difference between the reference signal and the feedback signal generated by the loop. The resulting phase error is filtered by the low-pass loop filter, which attenuates high-frequency components while preserving the low-frequency dynamics required for synchronization. The NCO generates a digital output signal whose frequency and phase are determined by a Frequency Control Word (FCW). The output frequency is obtained by incrementing a phase accumulator at each clock cycle, and the overflow rate of the accumulator defines the resulting frequency. Because the NCO operates at a frequency different from that of the reference signal, a frequency divider is included in the feedback path to scale down the oscillator output before phase comparison.

### 3.2. MEMS Accelerometers

An accelerometer can be modeled as a mass-spring-damper system, in which the displacement of the proof mass is proportional to the applied acceleration. The most widely used accelerometers are based on the piezoelectric principle or capacitive sensing [40]. Piezoelectric accelerometers generate an electrical signal in response to mechanical stress applied to piezoelectric materials, whereas capacitive accelerometers measure variations in capacitance caused by the displacement of a suspended mass. For this study, three commercially available digital MEMS accelerometers that support external or internal sampling synchronization were identified and are summarized in Table 1.

Analog Devices’ ADXL355 was selected for this research for its high-resolution, low-noise performance and digital interface [41]. The ADXL355 is a triaxial MEMS accelerometer with a digital output, widely used in geophysical and seismic instrumentation [42], structural health monitoring [43], inertial switching applications [44], and vibration and condition monitoring [45,46]. Its low power consumption and integrated digital communication interface make it suitable for embedded sensing systems and distributed sensor networks.

The ADXL355 has three high-resolution Σ–Δ Analog-to-Digital Converters (ADCs), providing 20-bit digital output for each axis. It supports three selectable measurement ranges, each associated with a corresponding sensitivity level, and integrates programmable digital filtering before and after the ADC stage to improve signal quality. In addition, the Output Data Rate (ODR), filter bandwidth, and cutoff frequency can be configured through internal registers. Communication with external devices is supported through both I^2^C and SPI protocols.

Accelerometers are susceptible to electrical noise, which can degrade measurement accuracy and reliability. Noise is any unwanted deviation from the true acceleration signal and may arise from thermal fluctuations, electronic interference, or quantization introduced during signal conditioning. In MEMS accelerometers, the dominant noise is Additive White Gaussian Noise (AWGN), which has a flat power spectral density and a Gaussian amplitude distribution in the time domain. Manufacturers typically specify the broadband noise as a Root Mean Square (RMS) value, expressed in µg (where g represents the g-force) or µV, measured under zero-acceleration conditions [47]. The RMS noise, $\eta_{RMS}$, is given by Equation (1).

(1) $$\eta_{RMS}=\rho \sqrt{1.6B}$$

where ρ is the sensor noise density, specified as 25 µg/$\sqrt{\mathrm{Hz}}$, and *B* is the cutoff frequency of the accelerometer filter. For an ODR of 1 kHz, the ADXL355 uses a cutoff frequency of 250 Hz. Substituting these values into Equation (1) yields an RMS noise of approximately 500 µg. Assuming Gaussian-distributed noise, the peak-to-peak noise can be statistically estimated as approximately $6.6\times \eta_{RMS}$ [48]. Accordingly, the estimated peak-to-peak noise is approximately 3.33 mg. In addition, the ADXL355 exhibits good thermal stability and low long-term drift, making it well-suited for applications requiring high measurement precision and repeatability [41].

### 3.3. Accelerometer Synchronization Using the ADXL355

Time synchronization is essential in distributed accelerometer networks, where multiple spatially separated sensors must acquire data simultaneously. Integrating and analyzing data from multiple accelerometers is challenging because precise temporal alignment is required to ensure data consistency. Furthermore, synchronization accuracy can be affected by both deterministic errors, such as sensor delays, signal propagation delays, and clock offsets, and random errors, including clock drift and measurement noise [24,25].

By default, the ADXL355 uses its internal clock to generate output data at a fixed ODR, eliminating the need for an external timing reference [41]. This configuration is suitable for applications in which asynchronous sampling is adequate. However, for applications that require synchronized sampling across multiple sensors, the ADXL355 supports synchronization through external signals, enabling simultaneous data acquisition.

The ADXL355 supports two external synchronization modes: interpolation-based synchronization and full external synchronization [41]. Interpolation-based synchronization simplifies the synchronization process but introduces additional delay and increased signal attenuation. In contrast, full external synchronization aligns the accelerometer’s operation with an external master clock (Ext_Clk) and an external synchronization signal (Ext_Sync), providing deterministic sampling relative to the external timing reference. In the proposed system, full external synchronization is used to align the ADXL355 sampling instants with the GPS-disciplined timing reference generated by the FPGA.

In full external synchronization mode, the Ext_Clk signal replaces the internal clock as the timing reference for the accelerometer. All internal functional blocks operate synchronously with Ext_Clk, while the Ext_Sync signal aligns the decimation filter output with a clock edge [41]. This mode is therefore well suited to distributed accelerometer systems that require simultaneous sampling. According to the accelerometer specifications, proper operation requires that: (1) Ext_Clk operate at 1.024 MHz; (2) an Ext_Sync pulse be applied to synchronize the internal ODR filters; and (3) the Ext_Sync pulse width be at least four periods of Ext_Clk.

If the Ext_Sync signal is removed, the ADXL355 automatically resumes operation using its internal timing reference [41]. In the proposed configuration, the Ext_Clk signal is applied to the INT2 pin, and the Ext_Sync signal is applied to the Data Ready (DRDY) pin. Samples acquired before the first synchronization pulse are discarded, allowing the ODR and its associated digital filters to realign with the external timing reference to ensure deterministic sampling.

**Figure 1:**
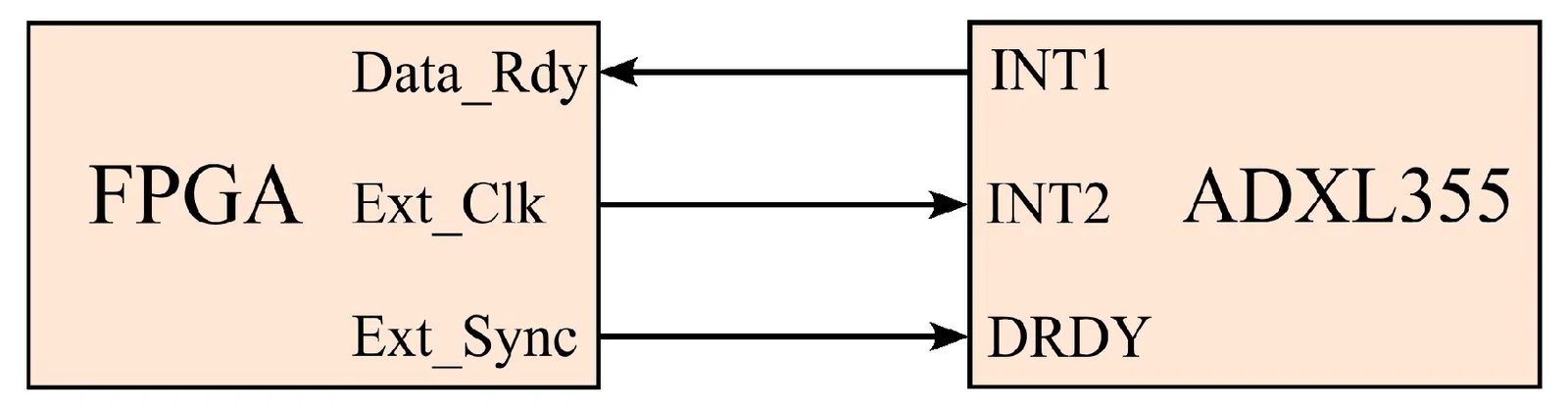
Signal interface between the ADXL355 and the FPGA.

The INT1, INT2, and DRDY pins of the ADXL355 are connected to the Data_Rdy, Ext_Clk, and Ext_Sync signals of the FPGA, respectively, as shown in Figure 1. During external synchronization, the DRDY pin receives the Ext_Sync pulse to align the internal ODR filters with the external timing reference, while the INT1 pin is asserted whenever a new set of acceleration samples becomes available. To preserve synchronization, the proposed design must respond with minimal latency to avoid introducing additional delay into the acquisition process.

## 4. FPGA-Based GPS Synchronization and Acceleration Measurement System

This section describes the proposed FPGA-based synchronization and acceleration measurement system and its experimental setup. The synchronization stage consists of a DPLL and two frequency dividers, whereas the acceleration measurement stage includes two Finite State Machines (FSMs) and the I^2^C and UART communication protocols.

### 4.1. DPLL Design

The DPLL implemented in this work is based on the architecture proposed by Morris [49]. It follows the structure of a conventional DPLL, with a limiting function added after the PD. A primary clock signal, denoted by $f_{Clk}$, drives all functional blocks of the DPLL. An asynchronous reset signal, Rst, initializes the DPLL by resetting all internal modules to their default states.

The pulse generation block processes the 1PPS signal from the NEO-6M V2 GPS module. Because the 1PPS signal is asynchronous to the system clock ($f_{Clk}$), it must first be synchronized to the clock domain to reduce metastability. The signal is passed through a two-stage flip-flop synchronizer. This synchronizer preserves the timing characteristics of the 1PPS signal and provides a reliable timing reference.

The PD estimates the phase error between the 1PPS reference signal ($S_{x}$) and the internal synchronization signal ($S_{I}$) generated by the frequency divider. Based on this phase error, the FCW is updated to set the NCO’s output frequency. While a phase error remains between the reference and internal signals, the FCW is adjusted in discrete steps to reduce it. Once phase lock is achieved, the FCW remains constant, allowing the DPLL to maintain synchronization. The operation of the FCW is given by Equation (2).

(2) $$FCW\left[n\right]=\left\{\begin{array}{cc} FCW[n-1] & S_{x}\left[n\right]=S_{I}\left[n\right]\\ FCW\left[n-1\right]+FCW_{ADJ_{+}} & S_{x}\left[n\right]>S_{I}\left[n\right]\\ FCW\left[n-1\right]+FCW_{ADJ_{-}} & S_{x}\left[n\right]<S_{I}\left[n\right] \end{array}\right.$$

where n≥0. The phase error between $S_{x}\left[n\right]$ and $S_{I}\left[n\right]$ is measured using a counter driven by the system clock ($f_{Clk}=50$ MHz), generating a temporal resolution of 20 ns. A measured phase error of zero indicates that the transitions of both signals occur within the same rising edge of the clock. However, in practice, the measured phase error fluctuates around zero because of the inherent timing jitter of both the 1PPS signal and the system clock. Although the DPLL does not include a dead zone, stable behavior is achieved through the joint operation of the limiter and the α-filter, which attenuate small phase fluctuations before the FCW is updated.

The terms $FCW_{ADJ_{+}}$ and $FCW_{ADJ_{-}}$ denote the discrete adjustment values applied to the FCW to increase or decrease the output frequency of the NCO. These adjustments change the number of oscillator cycles generated per period of the external 1PPS reference signal. The nominal FCW, which sets the initial operating frequency of the DPLL, is computed using Equation (3).

(3) $$FCW_{0}=\frac{Ext\_Clk}{f_{Clk}}\times 2^{N}$$

In this design, N=32 bits (see Equation (3)), which provides a trade-off between frequency resolution, hardware resource utilization, and processing performance on the FPGA. Although increasing the accumulator word length to N=64 bits would improve the NCO frequency resolution, it would also increase hardware complexity and timing constraints. Furthermore, the synchronization accuracy is primarily limited by the intrinsic timing jitter of the 1PPS signal rather than by the NCO quantization resolution. Therefore, a 32-bit accumulator provides sufficient resolution while enabling an efficient and scalable FPGA implementation.

The limiter constrains the magnitude of the phase error, preventing excessive adjustment to the FCW. As a result, the NCO’s output frequency changes more smoothly, improving loop stability as the DPLL converges to the target frequency of 1.024 MHz.

The low-pass filter is implemented as a first-order alpha filter with coefficients that are negative powers of two, as given by Equation (4), where $\alpha =\frac{1}{2^{m}}$ and 1≤m≤32.

(4) y[n]=αx[n]+y[n−1]−αy[n−1]

The time constant, τ, is given by Equation (5), where $T_{s}$ is the sampling period and α determines the dynamic response of the filter.

(5) $$\tau =T_{s}\left(\frac{1-\alpha }{\alpha }\right)$$

In this work, $\alpha =\frac{1}{2}$, with m=1 and $T_{s}=1$ s, resulting in τ=1 s. This result yields a moderately fast dynamic response, attenuating high-frequency phase fluctuations caused by the 1PPS timing jitter while preserving the low-frequency components required for synchronization.

The NCO functions as an oscillator of the accumulator, iterating its value as defined by Equation (5). The rate at which the accumulator overflows determines the oscillator frequency.

(6) NCO[n]=NCO[n−1]+FCW[n]

The DPLL configuration values are defined to generate the external clock signal (Ext_Clk) required by the accelerometer. The discrete adjustment values are computed from the external timing signal frequency $f_{S_{x}}=1$ Hz, the main oscillator frequency $f_{Clk}=50$ MHz, and the desired NCO output frequency Ext_Clk=1.024 MHz, using Equations (7)–(9).

(7) $$\begin{array}{cc} FCW_{ADJ+} & =\frac{f_{S_{X}}}{f_{Clk}}\times 2^{32}\approx 85 \end{array}$$

(8) $$\begin{array}{cc} FCW_{ADJ-} & =-\frac{f_{S_{X}}}{f_{Clk}}\times 2^{32}\approx -85 \end{array}$$

(9) $$\begin{array}{cc} FCW_{0} & =\frac{Ext\_Clk}{f_{Clk}}\times 2^{32}\approx 87960930 \end{array}$$

These values ($FCW_{ADJ+}$, $FCW_{ADJ-}$, and $FCW_{0}$) are incorporated into the hardware design as generic parameters, allowing adjustments for different frequency requirements. Finally, a frequency divider reduces the NCO output, Ext_Clk, to match the frequency of the $f_{S_{I}}$ signal before the signal is fed back to the PD.

The fixed values of $FCW_{ADJ+}$ and $FCW_{ADJ-}$ enable convergence during the acquisition stage. However, as the DPLL approaches phase lock, the fixed adjustment step causes small oscillations around the zero-phase-error condition because of the NCO’s finite frequency resolution. Although smaller adjustment steps could further reduce the residual phase error, the fixed-step approach was adopted in this work to preserve the simplicity of Morris’s original DPLL architecture [49].

### 4.2. Design of the FPGA-Based Synchronization and Acceleration Measurement System

**Figure 2:**
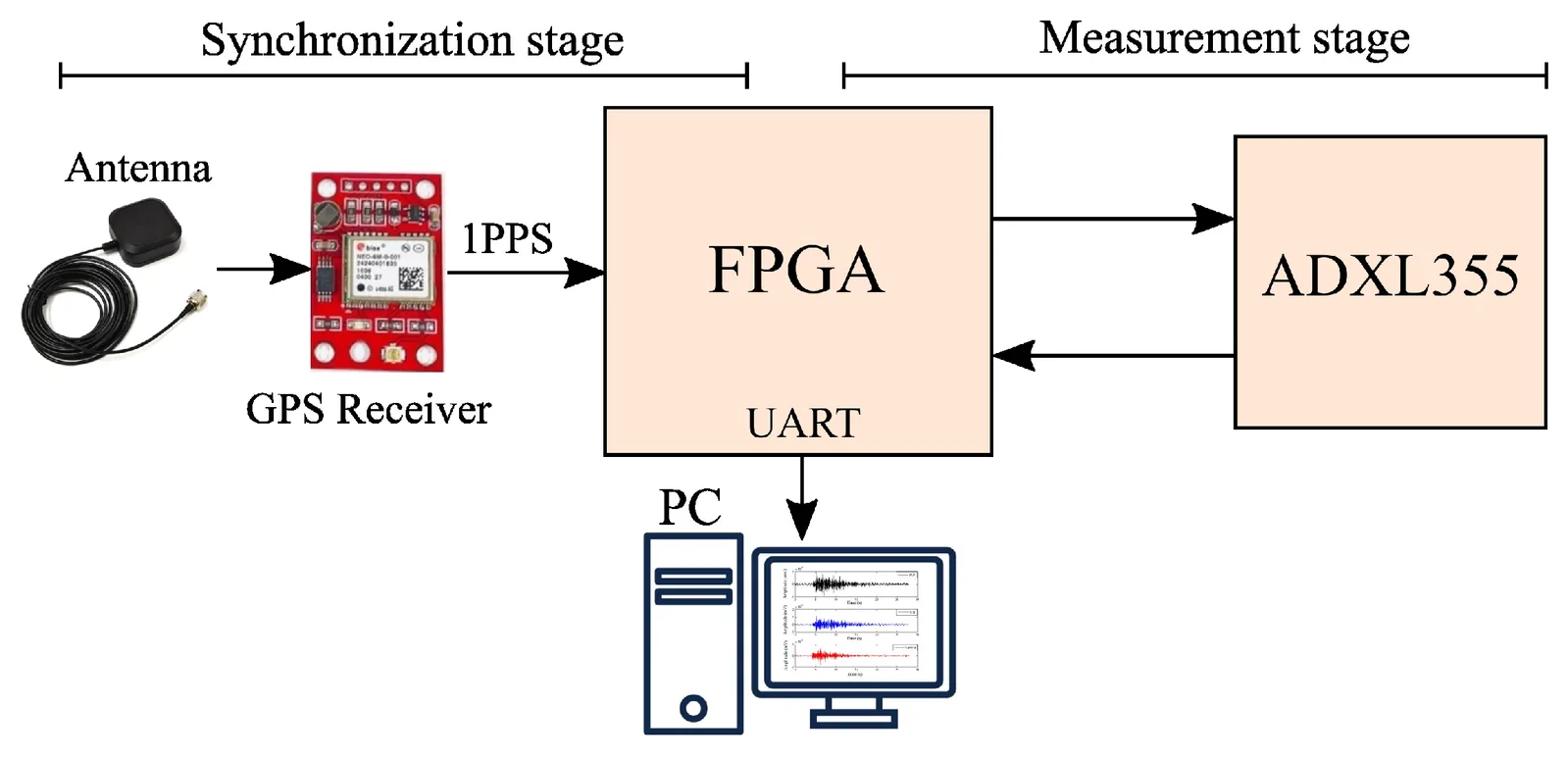
Overview of the proposed FPGA-based synchronization and acceleration measurement platform.

Figure 2 shows an overview of the proposed FPGA-based synchronization and acceleration measurement platform.

The synchronization stage minimizes the time difference between the rising edges of the 1PPS reference signal and the Ext_Sync synchronization signal. New acceleration samples are available on the rising edge of the INT1 signal.

During the data acquisition stage, acceleration samples are collected for subsequent analysis. Because the raw sensor output cannot be used directly for dynamic analysis, the acquired data are converted to acceleration values expressed in g-force. The ADXL355 stores acceleration measurements for the X, Y, and Z axes in three 20-bit registers: XDATA, YDATA, and ZDATA.

The register contents are left-justified, with bits arranged from most to least significant. The three registers can be transferred as a single data block, XDATA‖YDATA‖ZDATA, where ‖ denotes concatenation. Alternatively, data from an individual axis or a selected word length (8, 16, or 20 bits) can be read, depending on the application requirements.

#### 4.2.1. Synchronization and Acceleration Measurement System

**Figure 3:**
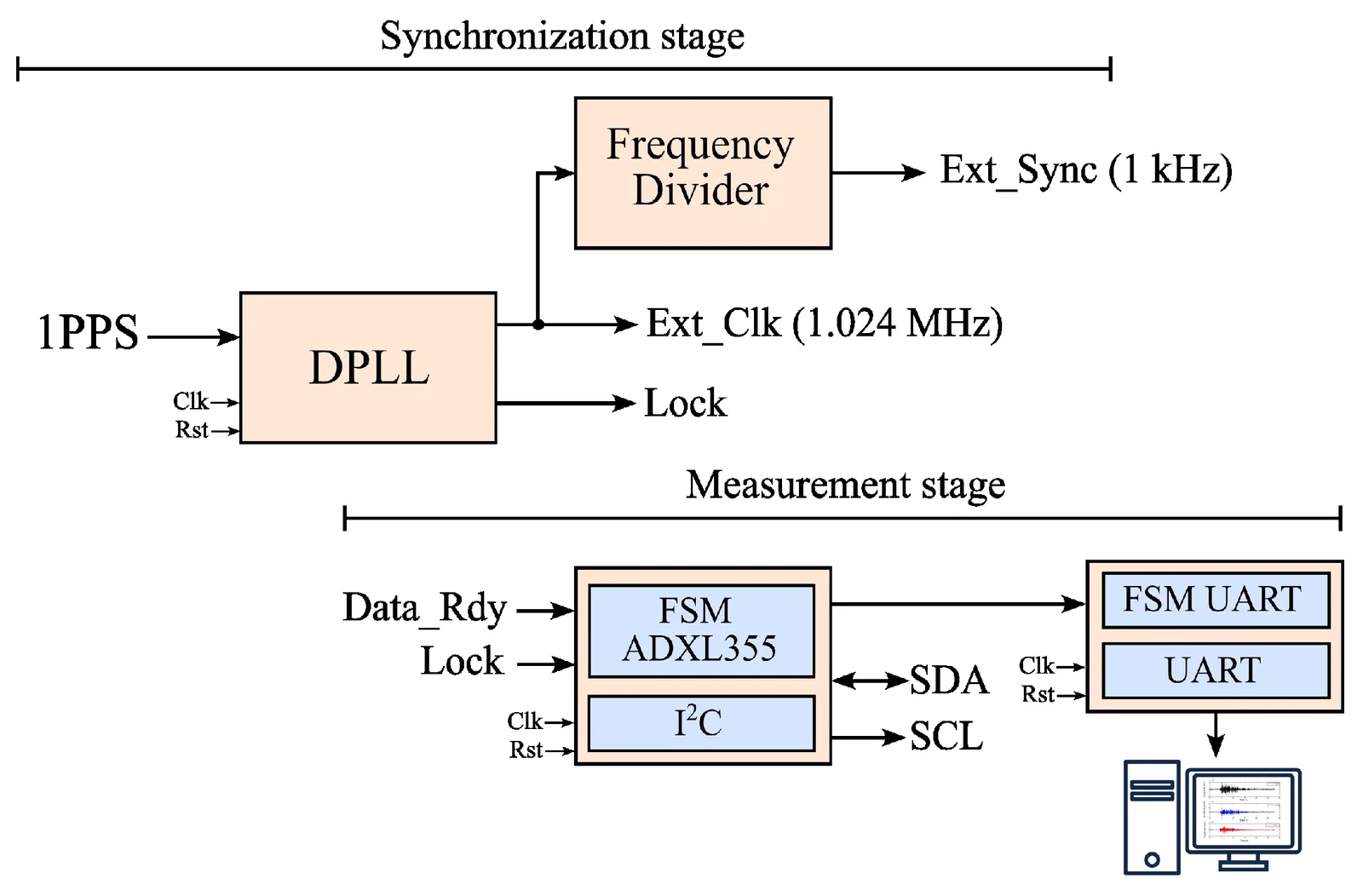
Functional block diagram of the FPGA-based synchronization and acceleration measurement (SAM) system.

Figure 3 presents the functional block diagram of the proposed FPGA-based Synchronization and Acceleration Measurement (SAM) system. The upper part of the figure depicts the synchronization stage, which includes a DPLL that generates a 1.024 MHz Ext_Clk signal synchronized with the 1PPS reference and a frequency divider that derives the 1 kHz Ext_Sync signal.

During synchronization, the DPLL aligns the 1PPS reference with the Ext_Clk signal, which serves as the external master clock for the ADXL355. The Ext_Clk signal synchronizes the accelerometer’s internal ODR, and acceleration samples from the three sensing axes are acquired synchronously.

The lower part of Figure 3 illustrates the acceleration measurement stage. It comprises an FSM for accelerometer control, an I^2^C master for sensor communication, a UART interface for data transmission, and an additional FSM that manages UART communication. All control, configuration, and data acquisition tasks are performed entirely on hardware.

The accelerometer’s FSM performs the complete initialization sequence required for synchronized operation. This includes enabling full external synchronization, setting the ODR to 1 kHz, selecting the ±2.048 g measurement range, enabling the INT1 data-ready interrupt, and starting continuous data acquisition. When the Lock signal is set, indicating that the DPLL has synchronized with the 1PPS reference, the FSM continuously monitors the Data_Rdy signal and initiates a new acquisition whenever new acceleration data are available.

Acceleration measurements are obtained via the I^2^C interface by reading the XDATA, YDATA, and ZDATA registers. Each axis is represented as a 20-bit two’s-complement value distributed across three consecutive registers. The three bytes are concatenated to reconstruct the complete acceleration value, as expressed in Equation (10), where x∈{1,2,3}, m∈{X,Y,Z}, and n=20.

(10) $$mDATAx=-m_{n-1}*2^{n-1}+\sum_{i=1}^{n-2}a_{i}2^{i}$$

Each *m*DATA*x* represents a raw acceleration value. The values are transmitted to the PC through the UART interface without additional processing in the FPGA. During software post-processing, the raw binary values are interpreted as signed integers and converted to units of g-force using a scale factor of $3.90\times 10^{-6}$ g/LSC for the selected measurement range of ±2.048 g, allowing their use for visualization and subsequent analysis.

#### 4.2.2. Synchronization and Acceleration Measurement with Synchronization Error Estimation

**Figure 4:**
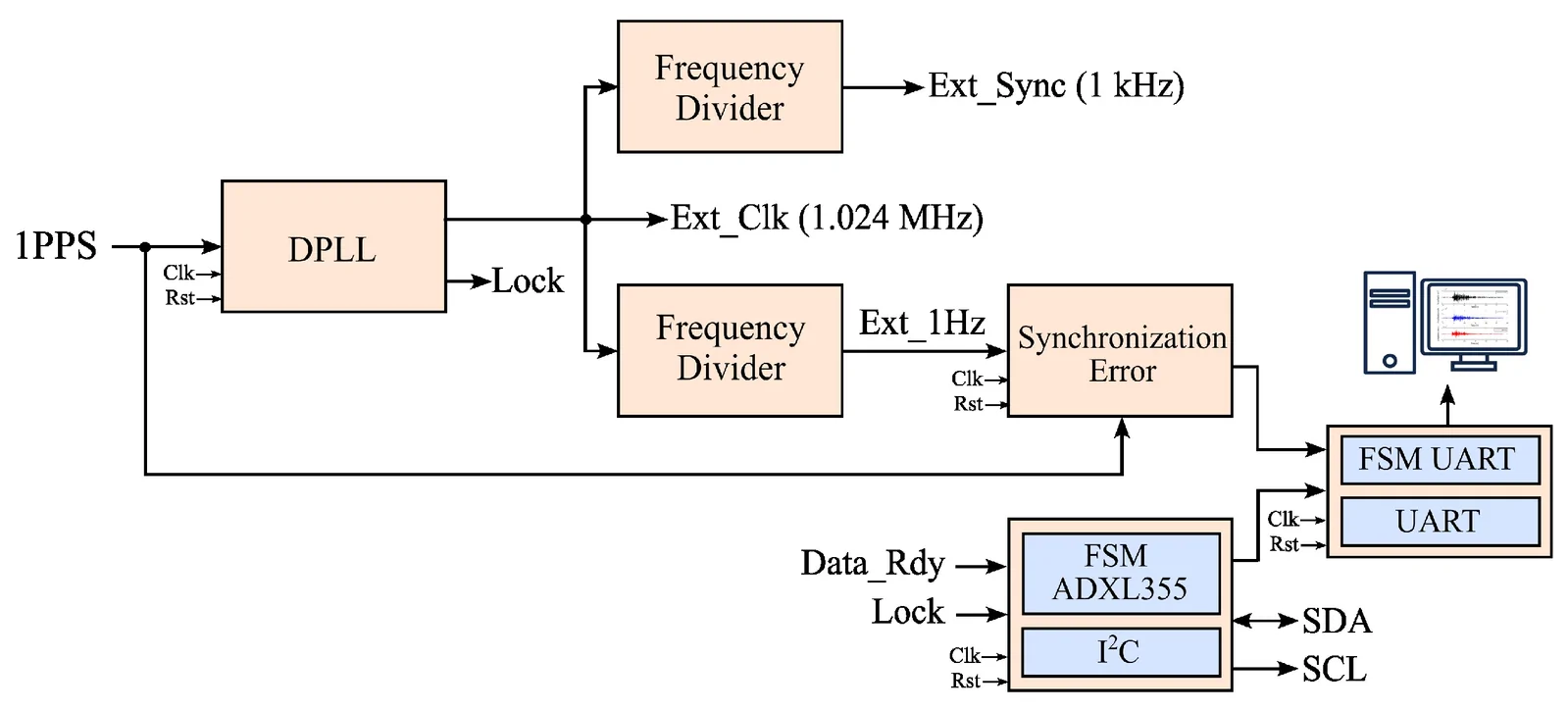
Functional block diagram of the FPGA-based synchronization and acceleration measurement, including synchronization error estimation (SAME).

Figure 4 shows the functional block diagram of the proposed FPGA-based Synchronization and Acceleration Measurement with Synchronization Error Estimation (SAME) system. This stage estimates the absolute offset of the synchronization time between the 1PPS reference signal and the Ext_1Hz signal, which is generated using a frequency divider, by measuring the time interval between the rising edges of both signals.

A counter driven by the 50 MHz system clock ($f_{Clk}$) counts the clock cycles between the two events. The measured time offset is obtained by multiplying the counter value by the system clock period (T=20 ns) and is transmitted to the PC via the UART interface.

Because both the Ext_1Hz and Ext_Sync signals are derived from the same Ext_Clk signal, the measured time offset between the 1PPS and Ext_1Hz signals is equivalent to that between the 1PPS and Ext_Sync signals. The proposed implementation provides the magnitude of the synchronization time offset; therefore, the measured values are non-negative. The measurement resolution is set by the 20 ns system clock period, and the reported time offset is expressed as an integer multiple of this resolution.

#### 4.2.3. Dual-Accelerometer Synchronization and Measurement System

**Figure 5:**
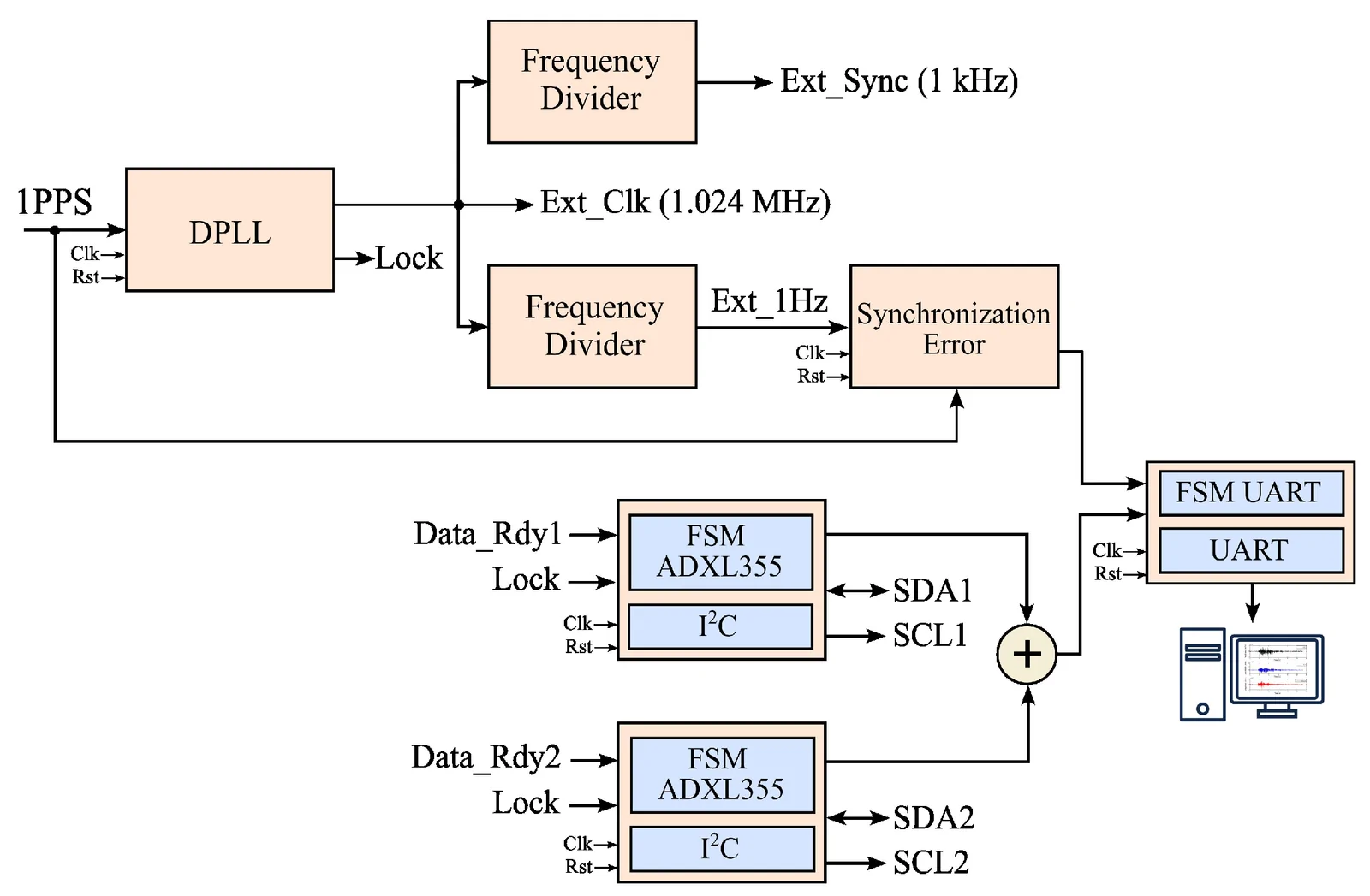
Functional block diagram of the FPGA-based Dual-Accelerometer Synchronization and Acceleration Measurement (D-SAME) system.

Figure 5 shows the functional block diagram of the proposed FPGA-based Dual Synchronization and Acceleration Measurement with Synchronization Error Estimation (D-SAME) system. The proposed architecture extends the single-accelerometer implementation to two synchronized ADXL355 accelerometers. Both sensors share the Ext_Clk and Ext_Sync signals, enabling simultaneous data acquisition from a common timing reference.

The accelerometers are mounted in opposing orientations, ensuring that identical mechanical vibrations yield acceleration measurements of opposite polarity (e.g., +g and −g along the same axis). As a result, the acceleration measurements can be summed (e.g., XDATA1 + XDATA2). Under ideal synchronization conditions and in the absence of external vibrations, this sum should be close to zero, providing a simple method to verify the consistency of measurements between the two accelerometers. Any residual variation from zero can be attributed to sensor mismatch or measurement noise.

### 4.3. Data Acquisition and Time Reference in FPGA

**Figure 6:**
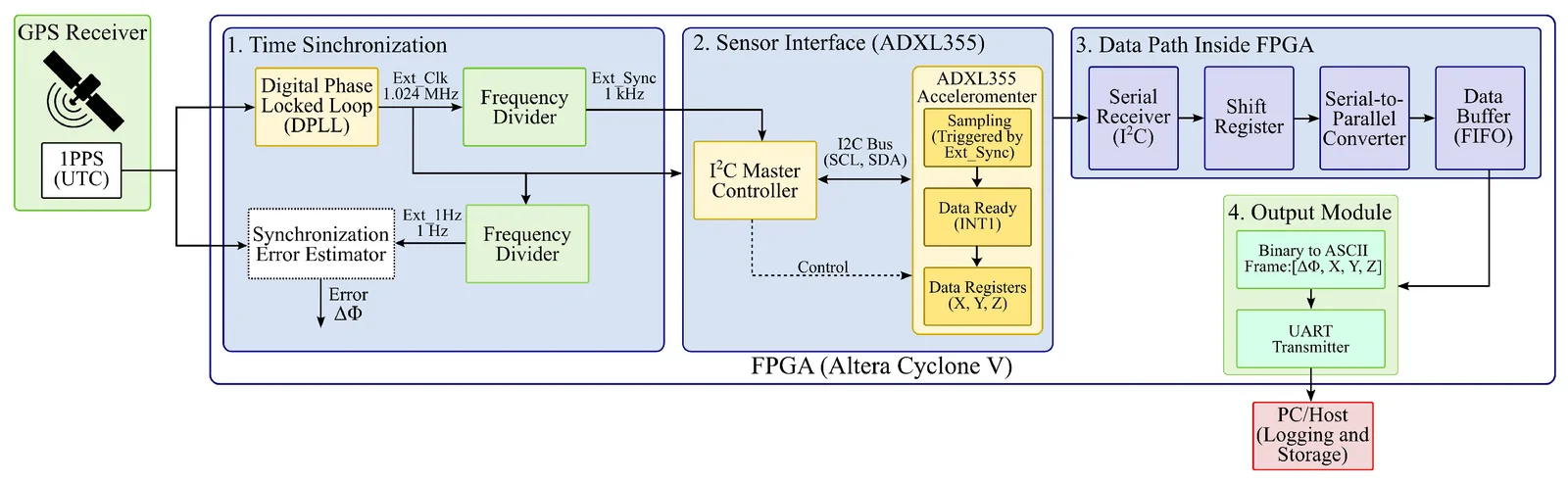
Internal functional blocks for a single acquisition node.

Figure 6 presents the internal timing and data-acquisition functions for synchronized acceleration measurement. The DPLL processes the 1PPS reference signal to generate the master clock Ext_Clk, from which the 1 kHz sampling signal Ext_Sync and the 1 Hz comparison signal Ext_1Hz are derived. The synchronization error is estimated from the 1PPS and Ext_1Hz reference signals. The Ext_Sync signal establishes the sampling time base of the ADXL355. The acquired acceleration data are subsequently read via the I^2^C interface by a controller. The received serial bytes are concatenated into parallel acceleration words and temporarily stored in a data buffer before being formatted and transmitted to the PC through the UART interface.

**Figure 7:**
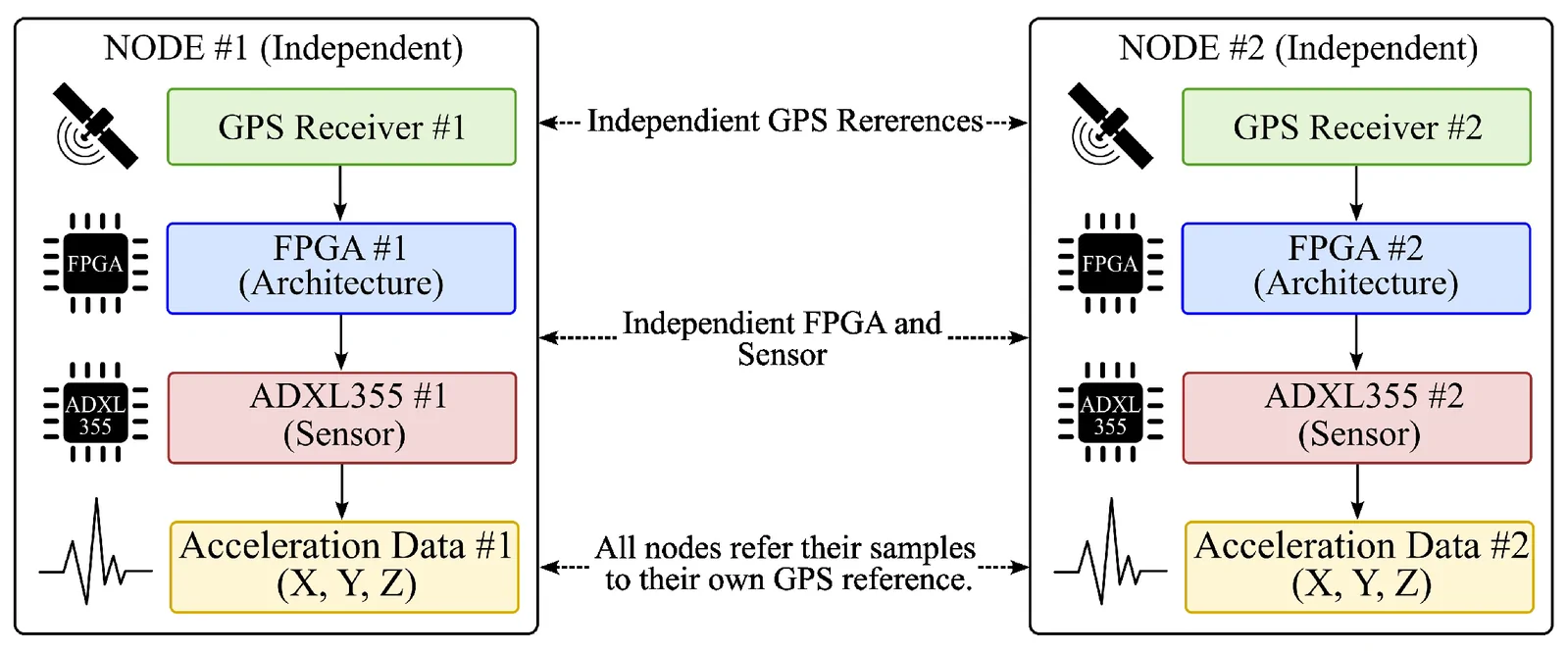
Architecture of two independent GPS-synchronized accelerometer nodes.

For distributed operation, each acquisition node has an independent GPS receiver and FPGA, as shown in Figure 7. Consequently, the nodes do not share a physical clock. Instead, each local Ext_Sync signal is independently synchronized with its corresponding 1PPS reference signal. This architecture allows acceleration samples from different nodes to be referenced to a common time scale based on the 1PPS signal.

## 5. Experimental Setup

**Figure 8:**
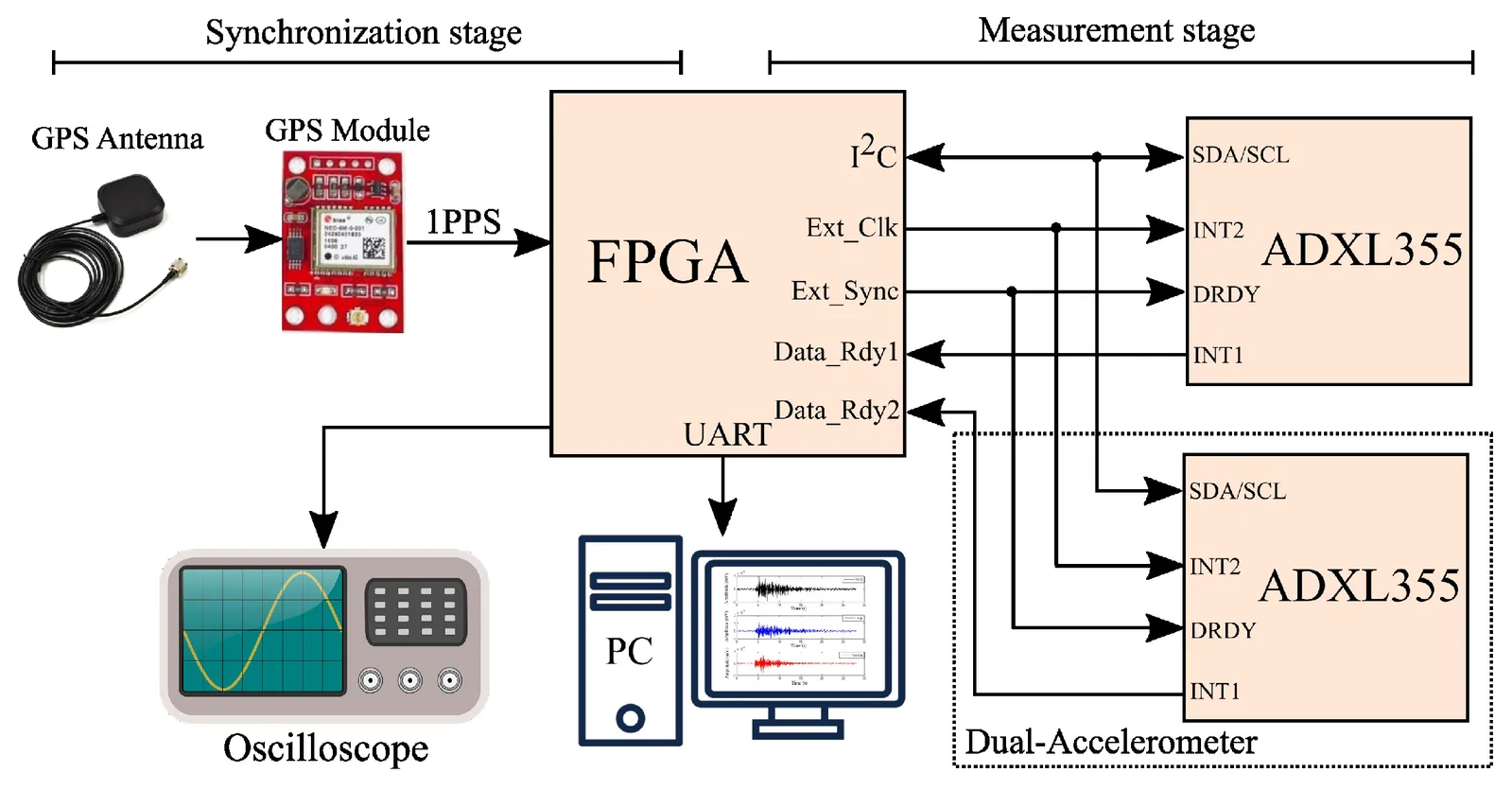
Experimental Setup of synchronization and acceleration measurement.

The experimental setup for the synchronization and acceleration measurement system is shown in Figure 8. It consists of a GPS antenna (Adafruit Industries LLC, New York, NY, USA), a NEO-6M V2 GPS receiver (u-blox Inc., Thalwil, Switzerland), an FPGA development board, one ADXL355 sensor (or two sensors for the dual-accelerometer configuration), a DS1054Z Rigol oscilloscope (Rigol Technologies Co., Ltd., Suzhou, China), and a PC. This setup was used to evaluate the performance of the synchronization by estimating the absolute synchronization time offset, acquiring three-axis acceleration measurements, assessing the effects of various environmental conditions on the accuracy of the synchronization, and recording accelerometer data in response to external vibrations.

**Figure 9:**
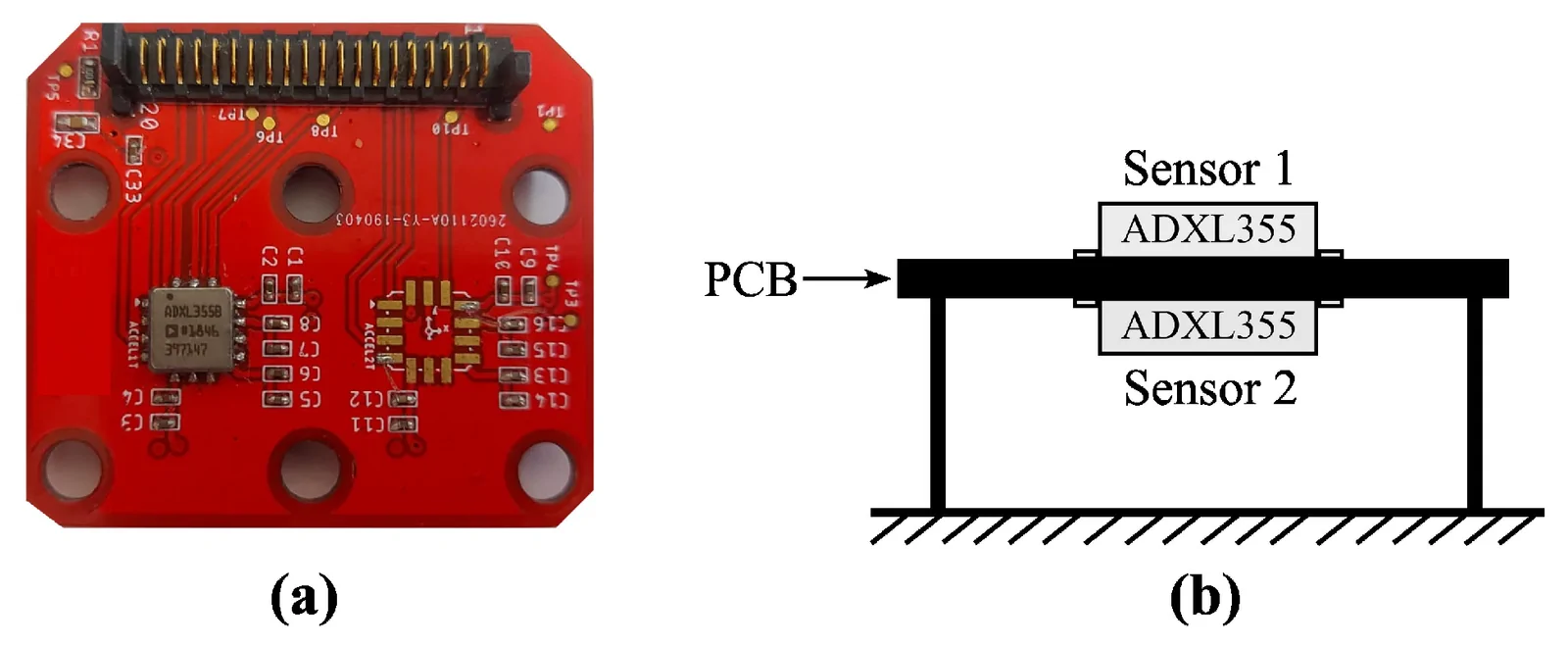
Sensor Kit: (**a**) Printed Circuit Board (PCB) layout, (**b**) sensor placement on the PCB.

The sensor kit, shown in Figure 9a, consists of two ADXL355 accelerometers mounted in opposite orientations along the *Z*-axis to produce acceleration measurements of opposite polarity under identical excitation. This arrangement allows acceleration measurements to be acquired from either a single sensor or both sensors simultaneously. Figure 9b shows a side view of the sensor kit positioned on a horizontal surface.

A common approach to validating the performance of an Inertial Measurement Unit (IMU) is to apply controlled dynamic excitation and evaluate the accelerometer’s frequency response. In this study, oscillatory motion at different excitation frequencies was generated using an SF-9324 Mechanical Wave Controller (PASCO Scientific, Roseville, CA, USA) driven by a PI-8127 function generator (PASCO Scientific, Roseville, CA, USA). The electrodynamic exciter produced sinusoidal vibrations proportional to the applied input voltage. The resulting acceleration measurements were used to characterize the frequency response of the proposed system for subsequent dynamic analysis.

**Figure 10:**
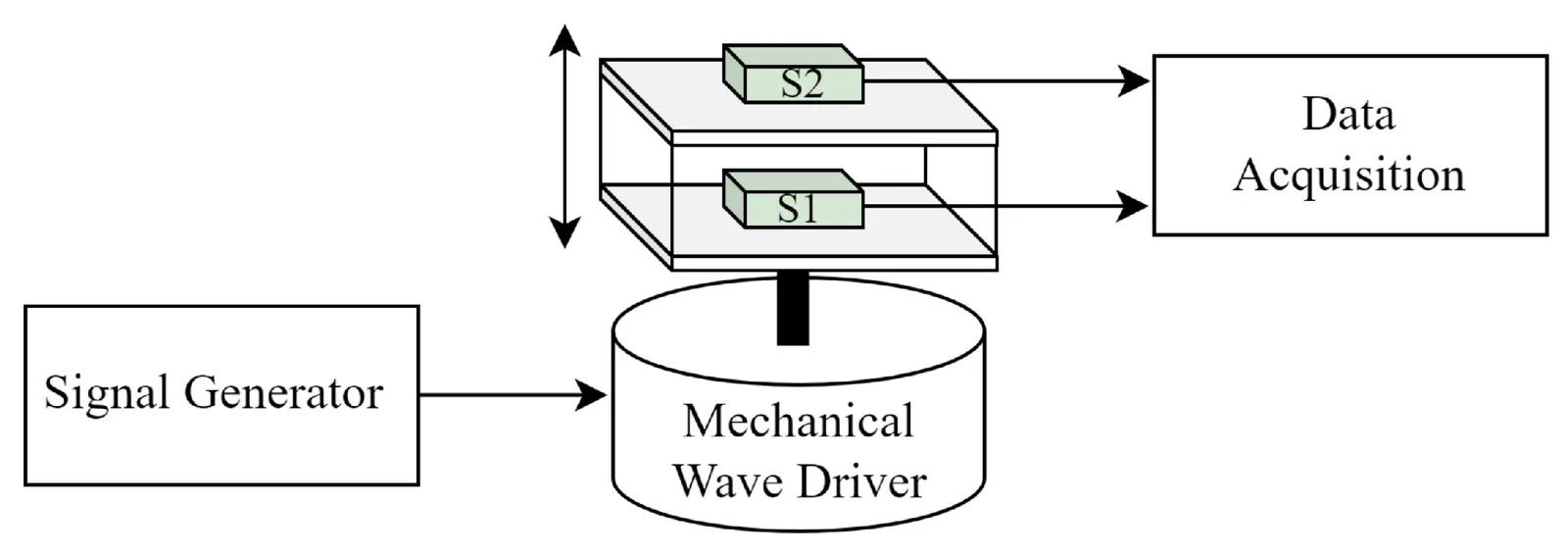
Experimental setup of the mechanical wave controller.

During the experimental evaluation, two accelerometers (S1 and S2) were mounted on separate support plates attached to the mechanical wave controller, as shown in Figure 10. Each accelerometer was connected to its own dedicated FPGA, which implemented an independent DPLL and was synchronized to its own GPS receiver through a dedicated 1PPS reference signal. Each FPGA generated its own Ext_Clk and Ext_Sync signals for sensor synchronization.

This configuration was selected to evaluate the proposed SAME system as an autonomous sensing node, representative of a distributed accelerograph network in which each station operates independently using its own 1PPS GPS reference. Care was taken to ensure the correct orientation and secure mounting of both accelerometers throughout the experiments. The experimental procedures and test conditions are described in detail in Section 6.4.

The joint calibration of the mechanical wave controller, the supporting mechanical structure, and the accelerometers was beyond the scope of this study and is planned for future research. Instead, the objective was to evaluate the overall performance of the proposed system under realistic operating conditions. Therefore, the measured frequency response reflects the combined effects of the vibration generator, the mechanical structure, and the two accelerometers. Although this approach provides a system-level assessment, it does not isolate the contribution of each component, which constitutes a limitation of the present study.

## 6. Experimental Results

This section presents the experimental validation of the proposed FPGA-based synchronization and acceleration measurement system. Section 6.1 reports the results of the FPGA synthesis and implementation. Section 6.2 and Section 6.3 present the synchronization performance and acceleration measurement results, respectively. Finally, Section 6.4 evaluates the frequency response of the proposed system under controlled dynamic excitation.

### 6.1. Synthesis Results and Performance

This section presents the FPGA implementation results for the proposed DPLL, SAM, SAME, and D-SAME architectures described in Section 4. In addition, the synthesis and timing performance metrics are compared with those reported in related studies. The designs were developed using a structural approach in VHDL and Verilog with Quartus Prime Lite v25.1, synthesized for a Cyclone V 5CSEBA6U23I7 FPGA, and experimentally validated on a DE10-Nano development board using the SignalTap II Logic Analyzer v25.1. The synthesis results are summarized in Table 2.

For the implementation evaluation, lookup tables (LUTs) and flip-flops (FFs) were used as resource utilization metrics, while the maximum operating frequency ($F_{max}$) and power consumption were used as performance metrics, as summarized in Table 2. The results show that integrating additional modules into the proposed architectures increases the utilization of hardware resources and power consumption while reducing the maximum operating frequency. This behavior is expected because the inclusion of additional synchronization, communication, and processing modules increases the logic depth and routing complexity of the FPGA implementation.

Table 3 compares the proposed architectures with related FPGA-based implementations in terms of device, hardware resource utilization (LUTs and FFs), maximum operating frequency ($F_{max}$), and power consumption. However, direct comparisons should be interpreted with caution because implementation results depend on several factors, including FPGA technology, device, design methodology, synthesis tools, communication protocols, and data transmission architecture. To the best of our knowledge, no previous FPGA-based implementation has combined GPS-referenced 1PPS synchronization, synchronized acceleration acquisition, and hardware-based synchronization error estimation within a single node.

This subsection provides a brief comparison with related FPGA-based implementations. Kumari et al. [17] interfaced an MPU6050 accelerometer with 16-bit resolution through the I^2^C protocol, whereas the proposed system employs a 20-bit ADXL355 accelerometer, providing higher measurement resolution. Perez et al. [18] interfaced an ADXL345 accelerometer with 13-bit resolution via the SPI protocol, with data transmitted in real time through Ethernet; in contrast, the proposed system incorporates GPS-referenced synchronization via the 1PPS signal to ensure synchronized acceleration acquisition. Tukiran et al. [19] employed an analog ADXL335 accelerometer with LabVIEW-based visualization and Ethernet communication, whereas this work utilizes a digital-output accelerometer, thereby reducing susceptibility to analog noise. Finally, Vita et al. [20] implemented FPGA-based acceleration measurement using a 65 nm CMOS standard-cell library, whereas the proposed system integrates GPS synchronization, acceleration measurement, and synchronization error estimation within a single FPGA-based autonomous sensing node.

### 6.2. Synchronization Results

This section presents the experimental evaluation of the proposed synchronization system using the setup shown in Figure 8. Three experiments were conducted:

1. Measurement of the Synchronization Error (SE), defined in this work as the absolute time offset between the 1PPS reference signal and the Ext_1Hz generated signal, together with the Sampling Delay (SD), calculated as the time difference between the Ext_1Hz and INT1 signals for three system configurations: a single accelerometer, two accelerometers on a single PCB, and two accelerometers on separate PCBs with independent FPGA-based synchronization units.
2. Evaluation of the synchronization error under three weather conditions (moderate rain, clear sky, and cloudy sky), followed by a one-way ANOVA.
3. Preliminary assessment of the frequency characterization of the Ext_Clk, Ext_Sync, and Ext_1Hz signals, together with an analysis of their frequency spectra.

#### 6.2.1. Measurement of SE and SD

**Figure 11:**
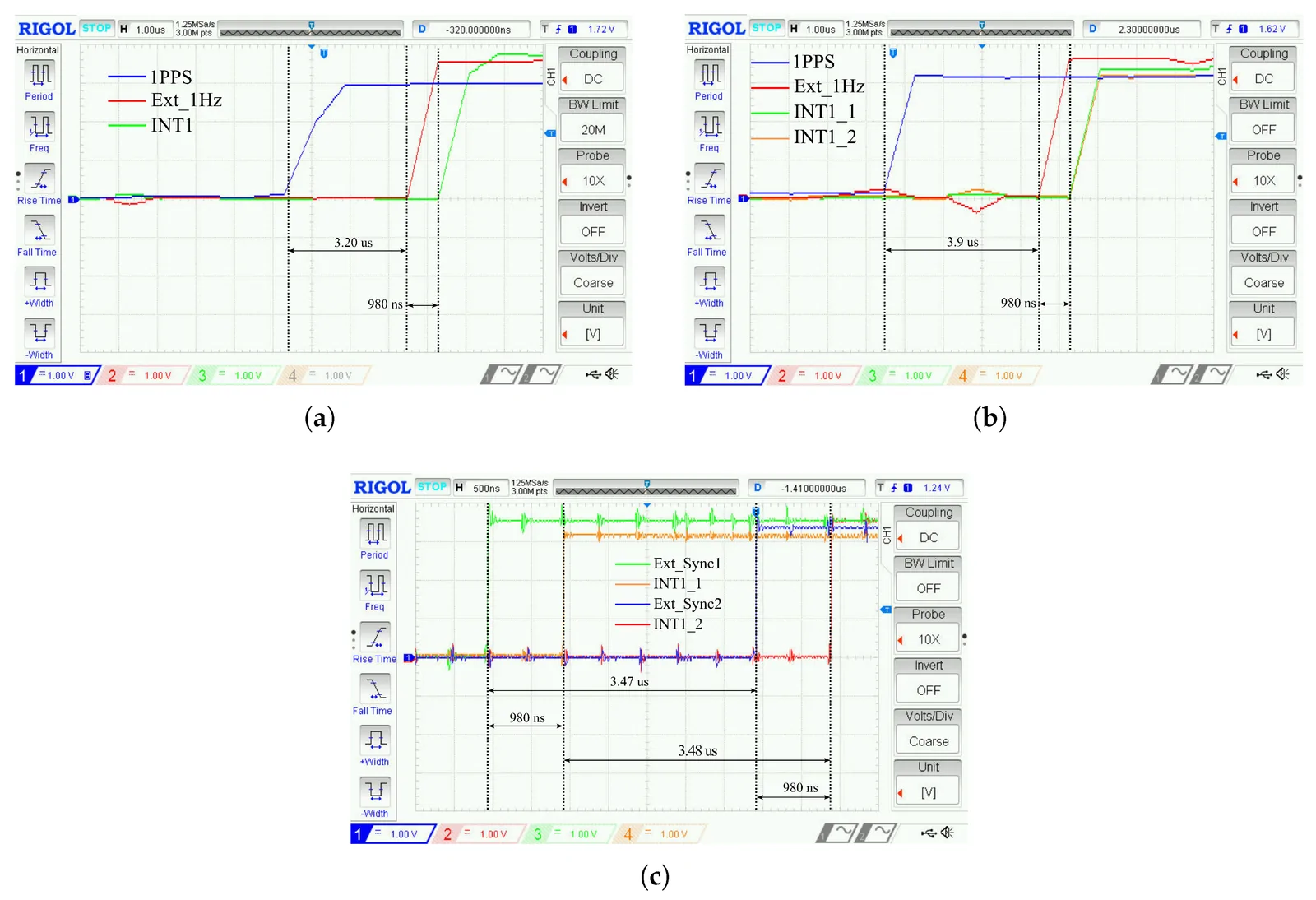
Measured synchronization error and sampling delay: (**a**) SAME system. (**b**) D-SAME system. (**c**) Two independent SAME nodes.

Figure 11a shows the measured 1PPS, Ext_Sync, and INT1 waveforms captured on the oscilloscope. The measured SE is 3.20 µs, while the SD is 980 ns. In particular, the SE varies because it includes the deterministic latency introduced by the digital synchronization chain, which comprises the DPLL output logic and the frequency divider. After lock is achieved, the residual phase offset remains bounded within a finite interval rather than converging to zero. This behavior results from the discrete FCW adjustment strategy and the finite timing resolution of the implemented DPLL, which provide stable frequency synchronization without guaranteeing absolute phase alignment.

Because both the Ext_Sync and Ext_1Hz signals are derived from the same Ext_Clk, they maintain a fixed phase relationship. Therefore, the SE measured between the 1PPS and Ext_1Hz signals is the same as that measured between the 1PPS and Ext_Sync signals. As shown in Figure 11a, the time interval between the rising edges of the Ext_1Hz and INT1 signals is approximately 980 ns, corresponding to one period of the 1.024 MHz external clock supplied to the ADXL355. Hence, this delay is an inherent characteristic of the synchronous sampling process rather than an additional synchronization error.

Figure 11b shows the measured 1PPS, Ext_1Hz, INT1_1, and INT1_2 signals. The outputs of INT1_1 and INT1_2 correspond to the data inputs of Data_Rdy1 and Data_Rdy2, respectively, as shown in Figure 8. In this configuration, a common Ext_Sync signal is distributed to both accelerometers. The SE is 3.90 µs, and the SD is 980 ns for both sensors. Despite the independent operation of the two INT1 outputs, the identical SD indicates that both accelerometers generate new acceleration data simultaneously with respect to the shared synchronization reference. This result confirms that the proposed D-SAME system preserves synchronous data acquisition across multiple accelerometers.

Figure 11c shows the measured Ext_Sync_1, INT1_1, Ext_Sync_2, and INT1_2 signals. Ext_Sync_1 and Ext_Sync_2 are generated on separate PCBs, each with an independent FPGA-based synchronization unit. Each system is synchronized with its own GPS receiver via a dedicated DPLL. Similarly, INT1_1 and INT1_2 are the interrupt outputs of each accelerometer.

The SD is 980 ns for both accelerometers, confirming that each SAME node preserves the deterministic sampling delay introduced by the ADXL355. The measured time difference between the Ext_Sync_1 and Ext_Sync_2 signals is 3.47 µs, indicating the synchronization offset between two independently synchronized sensing nodes. Consequently, the corresponding INT1 signals also differ by approximately 3.48 µs, while maintaining the same sampling delay relative to their respective local Ext_Sync signals.

These results demonstrate that the proposed architecture enables consistent operation across distributed measurement nodes. Although each node independently generates its synchronization signals using its own GPS receiver and DPLL, the resulting synchronization offset remains within the microsecond range, making the proposed system suitable for distributed acceleration measurement applications that require autonomous synchronization.

#### 6.2.2. Synchronization Error Measurement Under Different Weather Conditions

The timing accuracy of GPS-based synchronization may be affected by weather conditions that alter signal propagation between satellites and GPS receivers. Atmospheric disturbances, ionospheric variability, and geographical obstructions can introduce synchronization errors [50].

This section assesses synchronization errors caused by atmospheric disturbances. Weather conditions such as severe storms, heavy precipitation, cloud cover, and turbulence can degrade GPS signal quality by affecting signal propagation between satellites and GPS receivers. Understanding these effects is important for anticipating and mitigating synchronization errors.

Because GPS timing can be affected by atmospheric conditions, the robustness of the proposed synchronization system was experimentally evaluated under three weather conditions: cloudy sky, clear sky, and moderate rain. Three experimental configurations were considered:

1. Synchronization between the 1PPS signal and the FPGA-generated Ext_1Hz signal.
2. Synchronization between two independently generated Ext_Sync signals (Ext_Sync_1 and Ext_Sync_2), each produced by an autonomous FPGA synchronized with its own GPS receiver.
3. Synchronization between two independently generated Ext_1Hz signals (Ext_1Hz_1 and Ext_1Hz_2), each derived from a dedicated FPGA and a GPS receiver.

For each configuration, synchronization error measurements were collected over a 15-min interval at a sampling rate of 1 sample per second, resulting in 900 samples per experiment. A one-way ANOVA was conducted to determine whether the weather conditions produced statistically significant differences in the measured synchronization error. Cloudy-sky conditions were defined as complete cloud cover, clear-sky conditions as the absence of clouds, and moderate rain as precipitation with an intensity between 2 and 15 mm/h.

**Figure 12:**
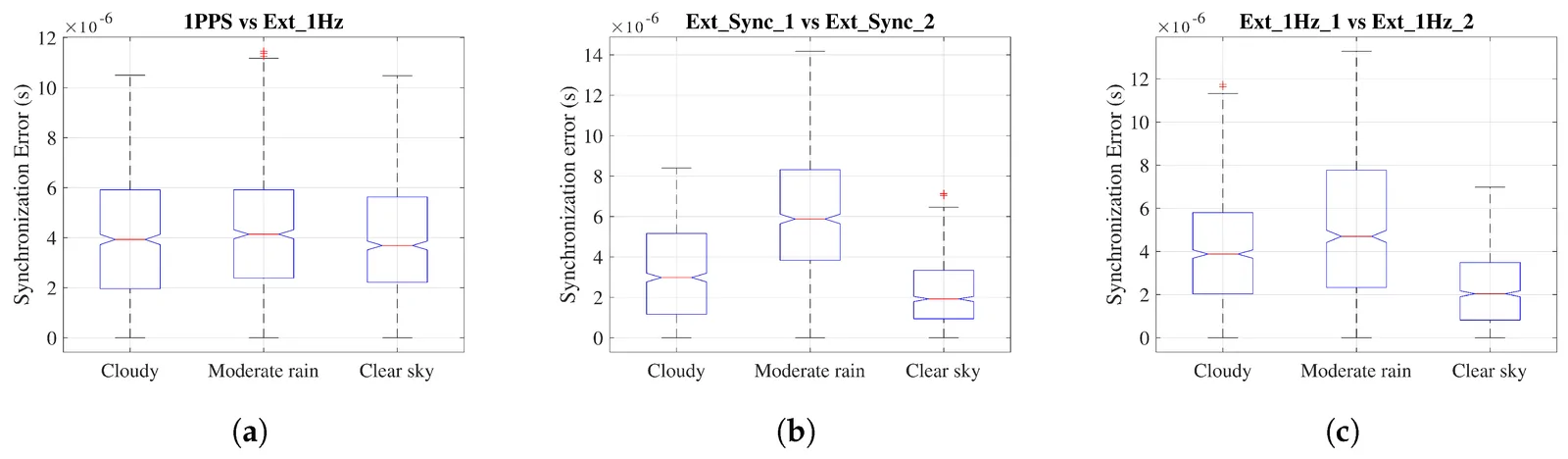
Box plot: (**a**) 1PPS vs. Ext_1Hz. (**b**) Ext_Sync_1 vs. Ext_Sync_2. (**c**) Ext_1Hz_1 vs. Ext_1Hz_2.

The results are presented in Figure 12 and in Table 4. The reported values are the absolute synchronization errors, defined as the absolute time interval between the rising edges of the signals (1PPS, Ext_1Hz, Ext_Sync_1, Ext_Sync_2, Ext_1Hz_1, and Ext_1Hz_2). Therefore, all synchronization errors are positive. Because this study evaluates the accuracy of the synchronization rather than phase lead or lag, only the magnitude of the synchronization error is reported.

For the first configuration, the synchronization error distributions across the three weather conditions are similar, as shown in Figure 12a. However, moderate rain produces the highest synchronization error, with a mean of 4.28 µs and a median of 4.14 µs (Table 4). In contrast, the lowest synchronization error occurs under clear-sky conditions, with a mean of 3.89 µs and a median of 3.69 µs.

For the second configuration, the lowest synchronization error occurs under clear-sky conditions, with a mean of 2.30 µs and a median of 2.04 µs, while moderate rain produces the highest synchronization error, with a mean of 5.18 µs and a median of 4.70 µs, as shown in Figure 12b and Table 4.

A similar trend is observed in the third configuration. As shown in Figure 12c and Table 4, moderate rain yields the highest synchronization error, with a mean of 6.14 µs and a median of 5.88 µs, while the lowest values occur under clear-sky conditions, with a mean of 2.21 µs and a median of 1.92 µs.

Overall, clear-sky conditions consistently yield the lowest absolute synchronization error across all three configurations. Although the absolute error does not preserve the sign of the phase offset, the results suggest that favorable atmospheric conditions reduce the timing uncertainty in GPS-based synchronization. In contrast, moderate rain is associated with a slight increase in the synchronization error.

An important factor in interpreting the previous results is the number of satellites visible to the GPS receiver under different atmospheric conditions. Under clear-sky, cloudy, and moderate-rain conditions, the receiver tracked up to 14, 12, and 9 satellites, respectively. This behavior is consistent with the receiver operating with only partial sky visibility (approximately half the sky), making it more susceptible to atmospheric attenuation and 1PPS signal blockage.

**Figure 13:**
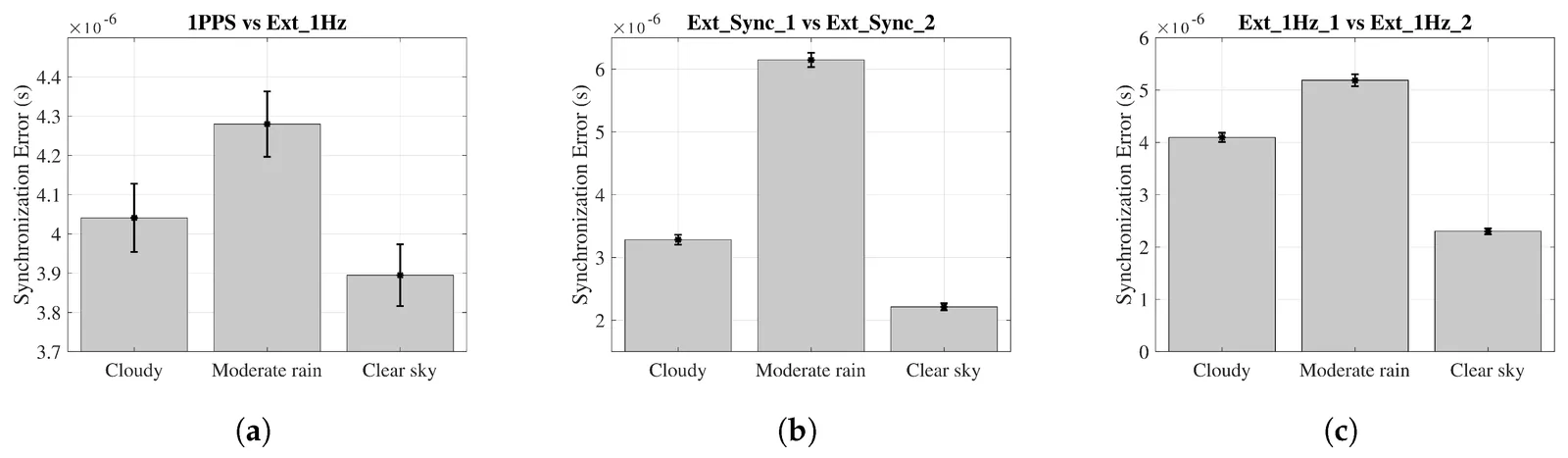
Error bars: (**a**) 1PPS vs. Ext_1Hz. (**b**) Ext_Sync_1 vs. Ext_Sync_2. (**c**) Ext_1Hz_1 vs. Ext_1Hz_2.

Figure 13 shows the error bars for the three synchronization configurations. In all cases, the standard errors are relatively small compared with the corresponding mean synchronization errors, indicating that the sample means are estimated with good precision. This result suggests that the observed differences between weather conditions are unlikely to be due to random sampling variability alone.

Table 5 presents the results of the one-way ANOVA, which complement the observations in Figure 12 and Table 4. The null hypothesis ($H_{0}$) assumes that the mean synchronization errors are the same across the three weather conditions for each experimental configuration. In contrast, the alternative hypothesis ($H_{1}$) assumes that at least one mean differs. Before performing the ANOVA, the normality assumption was verified to ensure the validity of the statistical analysis.

At the 95% confidence level, the ANOVA results indicate statistically significant differences between the three weather conditions for all experimental configurations (p<0.05). In particular, the second and third configurations produce very large *F*-statistics and *p*-values <0.001, providing strong evidence against $H_{0}$. These results suggest that atmospheric conditions influence the synchronization error, consistent with the reduction in the number of tracked GPS satellites observed under adverse weather conditions.

The synchronization experiments presented in this work were conducted outdoors under clear-sky, cloudy, and moderate-rain conditions. However, these measurements do not constitute a controlled characterization of thermal stability, since temperature was not independently controlled within a predefined operating range. Consequently, the results presented should not be interpreted as an exhaustive characterization of the thermal dependence of the synchronization error. Nevertheless, thermal variations affecting the oscillator and its associated electronic components may influence the synchronization error.

#### 6.2.3. Simulation Results

To verify the functional behavior of the proposed synchronization architecture, a simulation in ModelSim is included under ideal conditions. The simulation comprises the 1PPS, Ext_Clk, Ext_Sync, and Ext_1Hz signals, as well as the error signal (a positive decimal number of Clk clock pulses) used to estimate the synchronization error.

**Figure 14:**
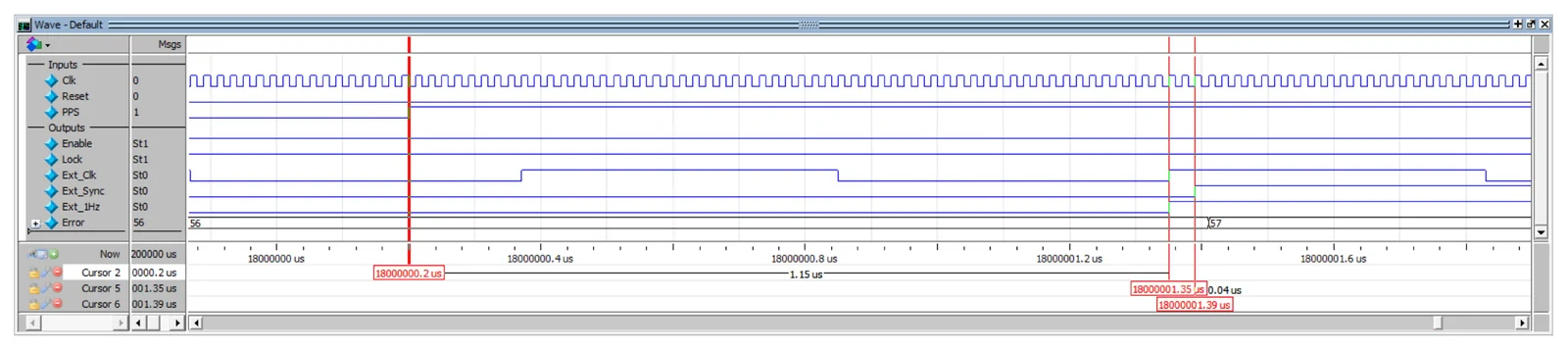
Functional simulation of the proposed synchronization architecture in ModelSim under ideal conditions.

Figure 14 shows the ModelSim simulation. The simulation results indicate that the synchronization error between 1PPS and Ext_1Hz is 1.15 µs (approximately 57 clock pulses), which falls within the range of the experimental results presented in Table 4. In addition, the time difference between 1PPS and Ext_1kHz is 1.154 µs. Based on the above, the results confirm the expected temporal relationship between the 1PPS reference signal and the internally generated clock signals.

Since the simulation was performed under ideal conditions, the results do not include or predict actual hardware errors or delays (jitter, noise, delays, among others). Therefore, the simulation is used primarily to verify the logical and temporal operation of the proposed synchronization architecture.

#### 6.2.4. Synchronization Accuracy, Timestamping, and Timing Considerations

The proposed architecture does not assign an absolute timestamp—for example, via the National Marine Electronics Association (NMEA) communication protocol—to each acquired acceleration measurement. In the current implementation, synchronization accuracy refers specifically to the temporal alignment between the 1PPS reference signal and the Ext_1Hz sampling signal. Consequently, the measured mean synchronization error of 4.07 µs represents the temporal alignment between the 1PPS reference and the Ext_1Hz signal and should not be interpreted as the end-to-end timestamp accuracy.

In a conventional quartz-based free-running sampling clock, the relative timing difference between independent nodes can progressively increase. In contrast, the proposed architecture corrects the timing base using a DPLL, thus limiting the accumulation of timing drift.

In a distributed seismic monitoring system, synchronization errors between nodes affect the temporal alignment, phase, and correlation of simultaneously acquired acceleration data; therefore, microsecond-level synchronization should be understood as a digital design capability rather than a requirement of the accelerometer itself.

Reducing the synchronization error to 10 µs or less reduces spatial uncertainty in seismic event localization, improves inter-station comparison, and limits drift among devices located at different points within a building. Some modern commercial accelerographs operate with errors close to or below 10 µs [35], while other high-end units achieve errors below 1 µs [34]. In practice, the achievable precision limit is determined more by ADC jitter, digital filter delay, and system latency than by the intrinsic accuracy of the PPS signal itself.

#### 6.2.5. Frequency Characterization of the Generated Timing Signals

The synchronization performance of the proposed system depends not only on the temporal alignment between the GPS 1PPS reference and the generated synchronization signals, but also on the frequency stability of the generated clocks. To quantify the deviation between the nominal and measured output frequencies, the frequency stability metric (ϵ), expressed in parts per million (ppm), is used, as defined in Equation (11). A lower value of ϵ indicates a smaller frequency deviation from the nominal value.

Therefore, a preliminary frequency characterization was performed on the Ext_Clk, Ext_Sync, and Ext_1Hz signals to evaluate the frequency deviation of the generated clocks from their nominal frequencies.

(11) $$\epsilon \left[ppm\right]=\frac{f_{out}-f_{nominal}}{f_{nominal}}\times 10^{6}$$

Therefore, a preliminary frequency characterization was performed on the Ext_Clk, Ext_Sync, and Ext_1Hz signals to evaluate the frequency deviation of the generated clocks from their nominal frequencies, as shown in Table 6.

Table 6 should be interpreted as a preliminary frequency characterization of the timing signals generated by the proposed synchronization architecture, not as a comprehensive frequency stability analysis. Because the Ext_Sync and Ext_1Hz signals are derived by digital frequency division from the Ext_Clk signal, they inherently share the same fractional frequency stability as the master clock. Consequently, the values reported for the three signals should not be treated as independent stability measurements, nor do they imply that the derived clocks exhibit intrinsically higher frequency stability than Ext_Clk. Instead, they represent the frequency estimates obtained from the measurement setup relative to the nominal output frequencies.

Characterizing the stability of the synchronization system would require isolating the contributions of the GPS 1PPS reference and the DPLL, which the measurement methodology used in this work cannot achieve. Such an analysis requires specialized instrumentation and time-domain stability metrics, such as Allan deviation or phase-noise measurements. Therefore, the present study focuses on verifying the correct generation of timing signals and the synchronization performance of the proposed architecture, while a comprehensive metrological characterization of the GPS receiver and the DPLL is considered a direction for future research.

For applications requiring higher long-term timing stability, the proposed architecture could be extended by incorporating higher-quality oscillators (TCXO/OCXO), multi-constellation GNSS synchronization, or holdover algorithms.

#### 6.2.6. Spectral Characterization of the Generated Timing Signals

**Figure 15:**
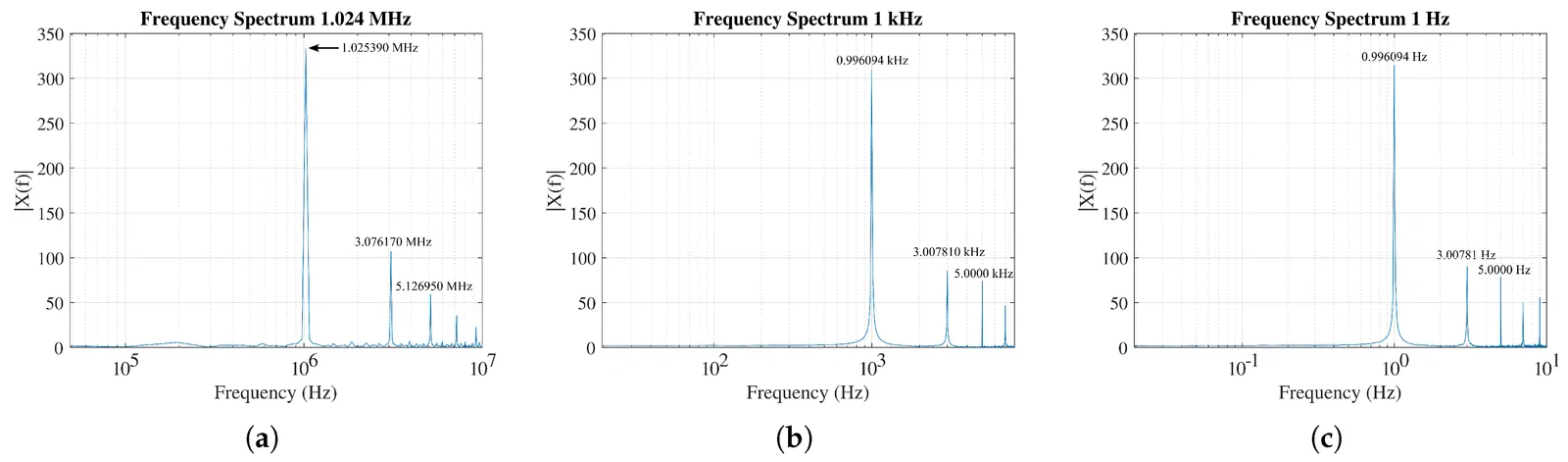
Frequency spectra of the generated signals: (**a**) Ext_Clk. (**b**) Ext_Sync. (**c**) Ext_1Hz.

Figure 15 presents the frequency spectra of the Ext_Clk, Ext_Sync, and Ext_1Hz signals obtained by the Fast Fourier Transform (FFT). The objective of this analysis is to verify the spectral content of the timing signals generated by the proposed synchronization architecture and to confirm the presence of the expected fundamental-frequency components. The FFT provides a spectral characterization of the generated signals but does not directly measure clock stability, timing jitter, or phase noise.

For the 1.024 MHz signal, the FFT was computed from 1200 samples acquired at a sampling frequency ($f_{s}$) of $f_{s}=50$ MHz, yielding a frequency resolution (Δf) of Δf=41.66 kHz and an acquisition time ($T_{acq}$) of $T_{acq}=2.4\times 10^{-5}$ s. For the 1 kHz signal, 1200 samples were acquired at $f_{s}=20$ kHz, Δf=16.66 Hz, and $T_{acq}=0.06$ s. Finally, for the 1 Hz signal, 1200 samples were acquired at $f_{s}=20$ Hz, Δf=0.01 Hz, and $T_{acq}=60$ s. In all cases, a rectangular window was applied.

In general, Figure 15 confirms the generation of the expected timing signals at 1.024 MHz, 1 kHz, and 1 Hz and illustrates their spectral characteristics under the adopted measurement conditions. A comprehensive characterization of clock stability, timing jitter, and phase noise would require dedicated metrological techniques, which are beyond the scope of this work.

### 6.3. Acceleration Measurement Results

**Figure 16:**
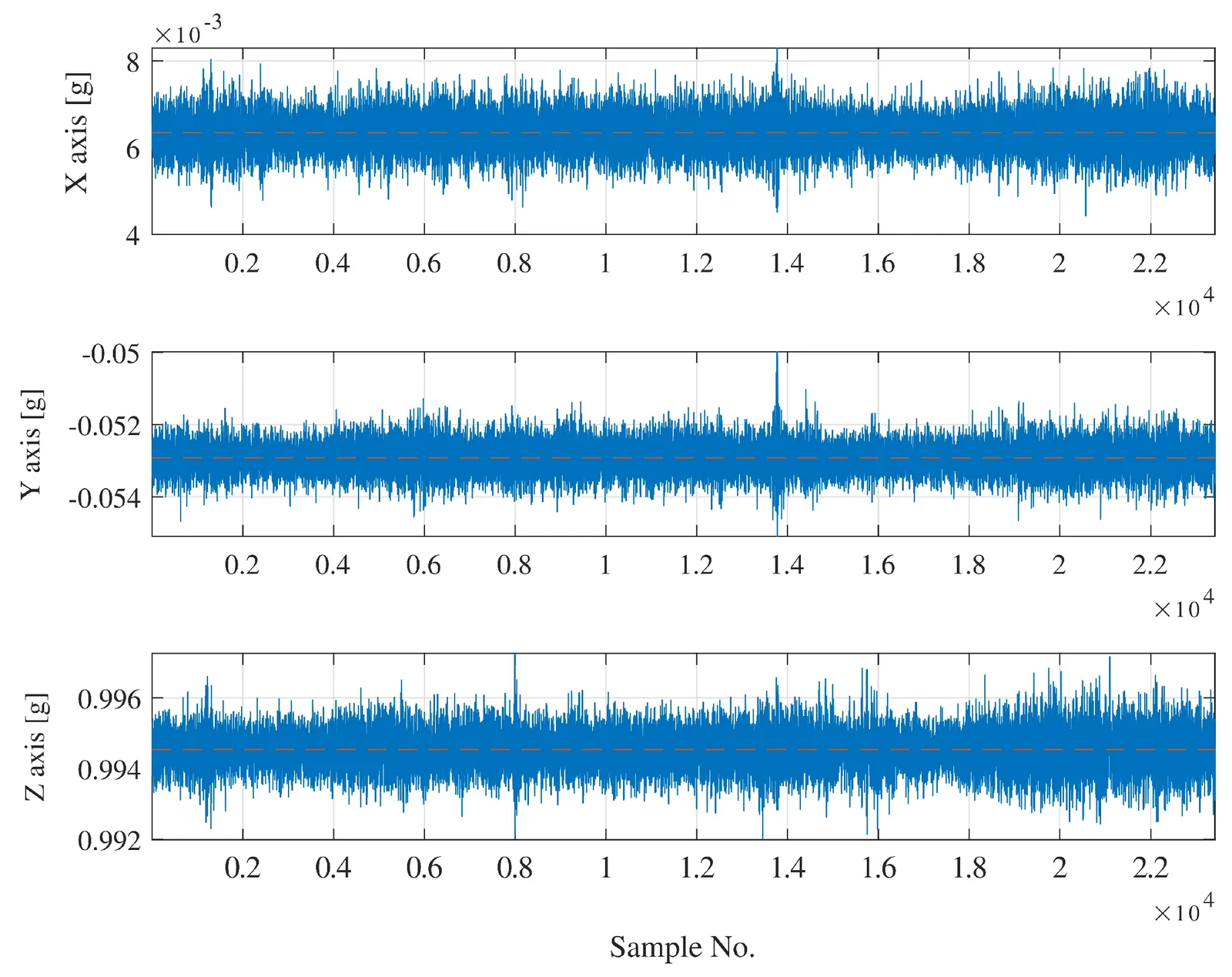
Acceleration data for the +Z axis orientation.

The acceleration output of a MEMS accelerometer exhibits small fluctuations even when the sensor is stationary. These variations arise primarily from the intrinsic sensor noise and measurement uncertainty, while the mean acceleration values may deviate slightly from their ideal values due to sensor offset. To characterize the static performance of the proposed system, acceleration data were collected for 60 s under resting conditions in a vibration-isolated environment. During the experiment, the sensor was mounted with its sensitive axis aligned with the positive +Z direction, as illustrated in Figure 16. Figure 16 presents the recorded acceleration along the three axes expressed in units of g-force. Under ideal static conditions, the expected acceleration values are 0 g for the *X* and *Y* axes and 1 g for the *Z* axis. The measured data show small deviations from these ideal values, along with low-amplitude random fluctuations attributed to sensor offset and measurement noise.

Five independent experiments were conducted to assess the repeatability of the measurement. Table 7 summarizes the standard deviations (Std. Dev.) of acceleration along the three axes, along with the mean acceleration along the *Z* axis. For test No. 1 shown in Figure 16, the measured standard deviations were 2.3447 mg, 1.6690 mg, and 3.1251 mg for the *X*, *Y*, and *Z* axes, respectively, whereas the mean accelerations were 15.0515 mg, −33.8569 mg, and 995.5329 mg along the *X*, *Y*, and *Z* axes, respectively. The low standard deviations observed across the five experiments indicate good repeatability and stable sensor operation under static conditions.

According to the ADXL355 datasheet, the sensor’s RMS noise depends on the selected ODR. The theoretical RMS noise ($\eta_{RMS}$) for the operating conditions used in this work was calculated using Equation (1). For an ODR of 1 kHz, corresponding to the Ext_Sync frequency used in the proposed synchronization architecture, the theoretical RMS noise is approximately 3.3333 mg. The experimentally measured standard deviations for the five experiments are in close agreement with this theoretical value, indicating that the proposed acquisition system preserves the intrinsic noise performance of the ADXL355 sensor without introducing significant additional measurement noise.

### 6.4. Frequency Response Results

The dynamic performance of the proposed synchronization and acceleration measurement system was evaluated according to the frequency-response requirements specified by the ISEE standard. These specifications define the acceptable transmissibility limits for an accelerograph by establishing amplitude-versus-frequency and phase-versus-frequency tolerance bands. An accelerograph is considered compliant if its measured response remains within these limits over the specified operating frequency range [51].

This section presents the experimental procedure used to evaluate whether the proposed system satisfies the frequency-response requirements specified by the ISEE standard. During each experiment, a data record consisting of 301,000 acceleration samples was acquired at $f_{s}=1$ kHz and $T_{acq}=301$ s. According to the Nyquist criterion, the maximum analyzable frequency was 500 Hz and $\Delta f=3.32\times 10^{-3}$ Hz.

For each experiment, one of nine predefined excitation frequencies (2, 3, 5, 10, 20, 40, 100, 150, or 175 Hz) and one of three peak-to-peak voltage amplitudes (0.2, 0.3, or 0.5 V) were selected using a function generator to drive the mechanical excitation system (see Section 5 and Figure 10). Two accelerometers (S1 and S2) were mounted on the vibration platform to record the resulting acceleration signals simultaneously. Each voltage amplitude was evaluated over the complete set of excitation frequencies before proceeding to the next voltage level. The accelerometers operated independently, each connected to a dedicated FPGA and synchronized by its own GPS receiver.

**Figure 17:**
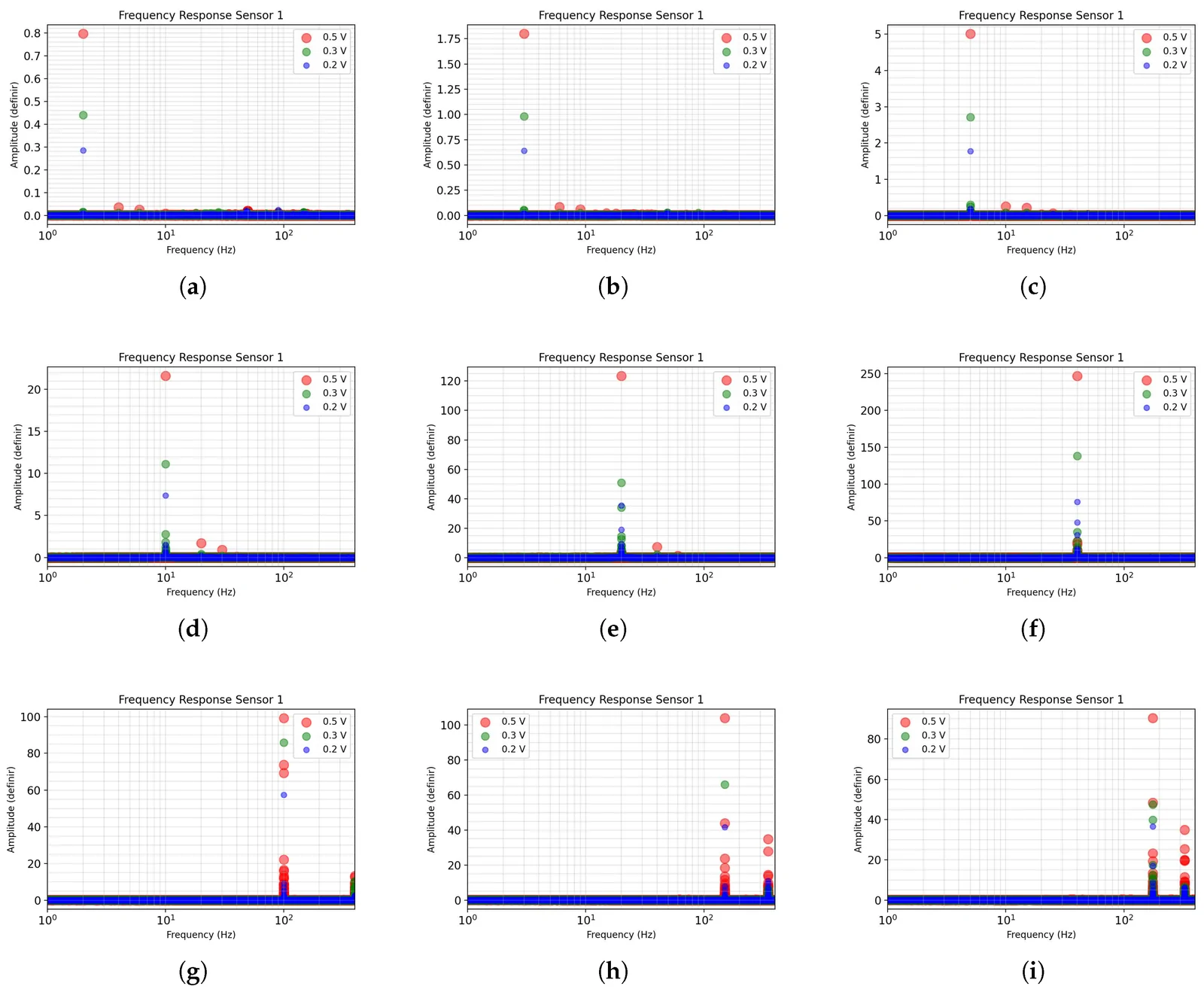
Frequency response of the accelerometer: (**a**) 2 Hz. (**b**) 3Hz. (**c**) 5 Hz. (**d**) 10 Hz. (**e**) 20 Hz. (**f**) 40 Hz. (**g**) 100 Hz. (**h**) 150 Hz. (**i**) 175 Hz.

Figure 17 presents the frequency response obtained from the acceleration measurements acquired by Sensor 1 under the experimental conditions described in Section 5. The sensor was evaluated at the nine predefined excitation frequencies and the three peak-to-peak excitation voltages. Figure 17a–i correspond to the excitation frequencies of 2, 3, 5, 10, 20, 40, 100, 150 and 175 Hz, respectively, with each subfigure showing the responses obtained for the three excitation voltage levels.

The results show a clear correspondence between the applied excitation frequency ($F_{i}$) and the dominant frequency component measured by the accelerometer ($F_{a}$). For all test conditions, the largest spectral amplitude occurs at the excitation frequency, confirming that the proposed synchronization and acquisition system captures the fundamental vibration component. Additional low-amplitude spectral components are observed. These components are attributed to the combined effects of the mechanical excitation system, structural resonances, environmental disturbances, and measurement noise, rather than the synchronization errors introduced by the proposed FPGA-based acquisition system.

**Figure 18:**
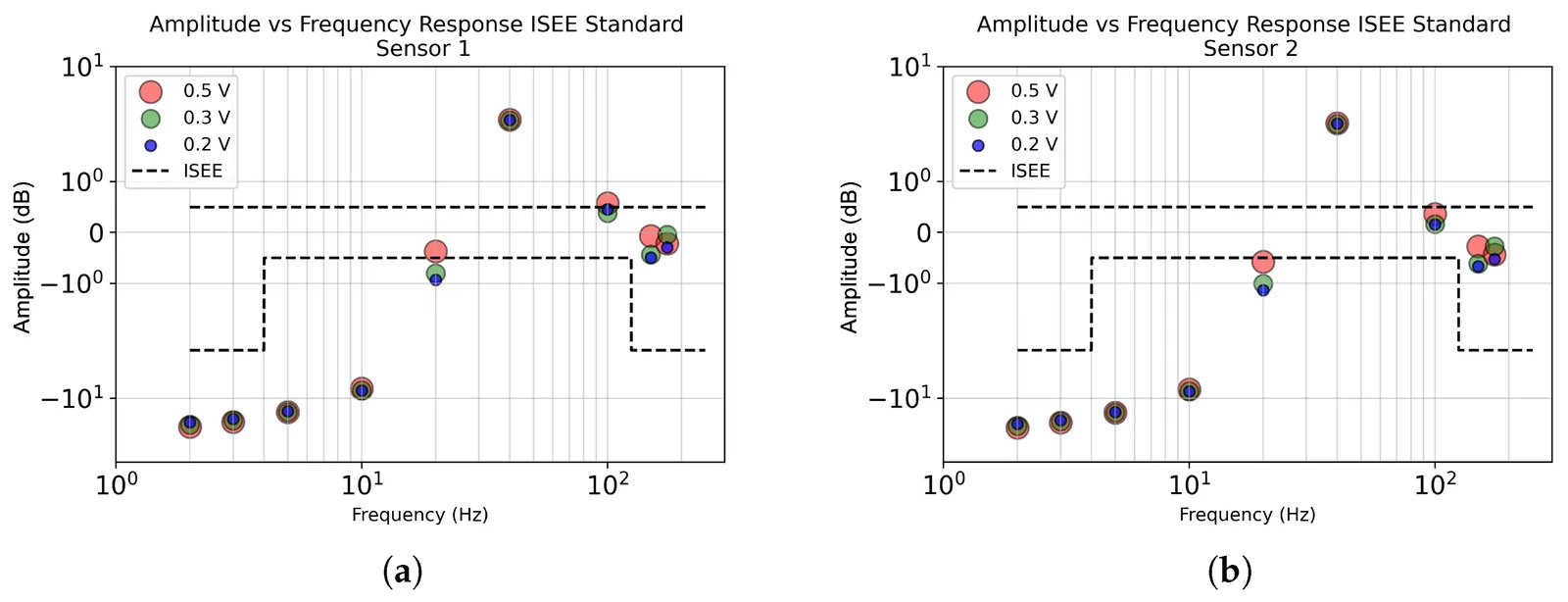
Measured gain-versus-frequency response compared with the ISEE amplitude tolerance limits of: (**a**) Sensor 1. (**b**) Sensor 2.

Figure 18 summarizes the frequency-response results from the experimental measurements and compares them with the amplitude tolerance limits specified by the ISEE standard. Figure 18a and Figure 18b correspond to Sensors 1 (S1) and 2 (S2), respectively. The results show that both sensors remain within the ISEE tolerance band over an intermediate frequency range of approximately 20–100 Hz. Within this interval, the measured gain closely follows the reference response defined by the standard.

At low frequencies ($F_{i}\leq 10$ Hz) and high frequencies ($F_{i}>100$ Hz), the measured responses deviate from the prescribed tolerance limits. These deviations may be attributed to the combined effects of the vibration exciter, the mechanical mounting structure, and the accelerometer dynamics, rather than solely to the sensor itself. Consequently, the results indicate that the proposed measurement system satisfies the ISEE amplitude-response requirements over a limited portion of the evaluated frequency range.

Furthermore, responses at excitation amplitudes of 0.2 V, 0.3 V and 0.5 V exhibit similar trends at all frequencies tested. This observation suggests that the measured frequency response is largely independent of the excitation amplitude within the evaluated range, indicating consistent system behavior across different excitation levels.

**Figure 19:**
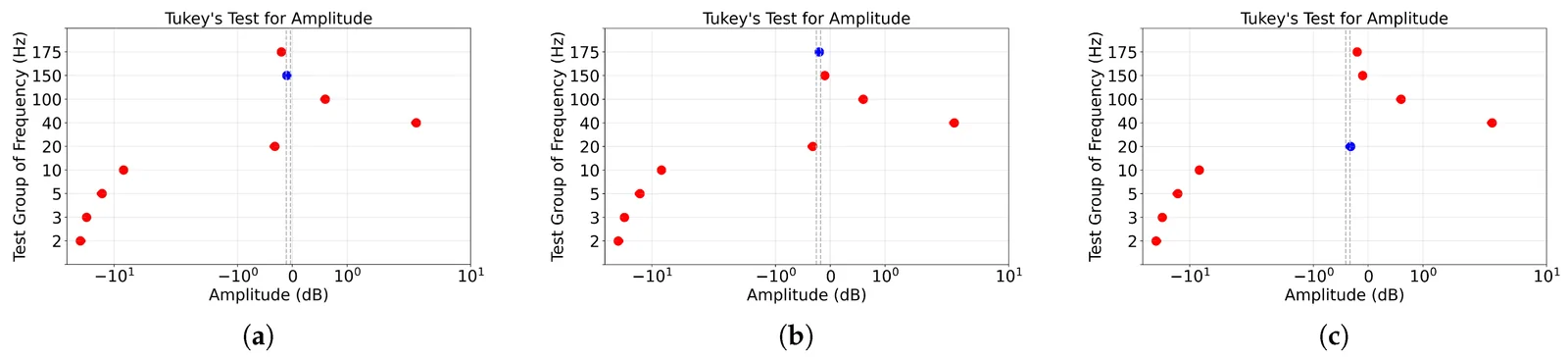
Tukey test analysis of gain vs. frequency response: (**a**) 0.2 V. (**b**) 0.3 V. (**c**) 0.5 V.

Figure 19 presents the results of Tukey’s post hoc multiple-comparison test, which complements the one-way ANOVA analysis by identifying differences in the measured gain among the nine excitation frequencies for each excitation voltage (0.2, 0.3, and 0.5 V) shown in Figure 17. The null hypothesis ($H_{0}$) assumes that the mean values of the estimated frequencies ($F_{a}$) recorded by the accelerometers are equal for the nine predefined frequency values ($F_{i}$). In contrast, the alternative hypothesis ($H_{1}$) suggests that these mean frequency values differ significantly.

Before conducting the statistical test, the normality of the data sets was verified. Based on the *p*-values obtained and a confidence level of 95%, the analysis confirms statistically significant differences among the estimated frequencies. This trend remains consistent across all evaluated amplitude values (0.2 V, 0.3 V, and 0.5 V). Under all three voltage conditions, the low frequencies maintain a gain close to 0 dB, indicating stability of the system. Additionally, at the highest voltage (0.5 V), an increase in dispersion is observed at high frequencies, suggesting a less controlled response or increased system sensitivity to disturbances.

Experimental validation was carried out using a 1 kHz sampling time base generated by the FPGA, without simultaneously acquiring identical transient shock signals from two independent ADXL355 sensors operating at their maximum output data rate (4 kHz). Consequently, measurement-level synchronization was not directly evaluated against real external vibration events.

## 7. Conclusions

This paper proposes an autonomous FPGA-based synchronization architecture for distributed accelerograph systems. Unlike previous FPGA-based implementations that focus on acceleration acquisition or data transmission, the proposed architecture synchronizes the entire acceleration measurement chain, from the GPS 1PPS reference to the sensor sampling instant, and simultaneously provides real-time synchronization error estimation on a single hardware platform.

Experimental validation showed a mean synchronization error of 4.07 µs between the GPS 1PPS reference and the internally generated 1 Hz synchronization signal. Additional experiments in clear-sky, cloudy, and moderate-rain conditions confirmed the reliability of the synchronization process. Frequency-response measurements also showed compliance with the ISEE amplitude-response requirements in approximately 40% of the evaluated frequency range.

A primary contribution of this work is evidence that autonomous sensing nodes, each equipped with an independent GPS receiver and an FPGA, generate locally disciplined synchronization signals and maintain synchronized acceleration acquisition without centralized clock distribution. This capability is an important step toward scalable distributed accelerograph networks for scientific instrumentation and for structural or seismic monitoring.

The proposed implementation requires 1163 LUTs, 606 FFs, and 133.78 mW, making it suitable for resource-constrained embedded platforms. Although this work provides an experimental validation of the proposed architecture, the frequency stability characterization presented herein should be regarded as a preliminary evaluation of the generated clock signals rather than a rigorous characterization of GPS or DPLL timing stability.

Future work will focus on reducing the synchronization error to below 1 µs, incorporating higher-stability oscillators (TCXO/OCXO), and performing comprehensive timing characterization using specialized metrics such as Allan deviation, phase-noise analysis, and jitter measurements. In addition, we intend to extend the proposed architecture by incorporating timestamping via the National Marine Electronics Association (NMEA) communication protocol, accounting for the delays associated with accelerometer data acquisition and readout. Finally, we propose to evaluate the system’s performance at vibration frequencies beyond those investigated in this study and to conduct long-term, temperature-dependent stability tests to assess synchronization accuracy across a range of outdoor operating conditions.

## Figures and Tables

**Table 1:** Comparison of Digital MEMS accelerometers with synchronized sampling capability.

Device	Range g	ADC Bits	Sensibility [µg/LSB]	NSD µg/$\sqrt{\mathrm{Hz}}$	Output Protocol
ADXL372 ^*a*^	±200	12	1000.0	150.0	SPI/I^2^C
ISM330IS ^*b*^	±2, ±4, ±8, ±16	16	61.0	70.0	SPI/I^2^C
ADXL355	±2.048 ±4.096 ±8.192	20	3.9 7.8 15.6	22.5	SPI/I^2^C

NSD: Noise Spectral Density. ^*a*^ Analog Devices Inc., Wilmington, MA, USA. ^*b*^ STMicroelectronics Inc., Geneva, Swiss.

**Table 2:** Synthesis results and performance metrics of synchronization and acceleration measurement designs.

Design	LUTs	FFs	$F_{\max}$ (MHz)	Power (mW)
DPLL (Morris [49])	422	208	119.76	129.78
SAM (Figure 3)	930	522	119.70	130.81
SAME (Figure 4)	1163	606	85.83	133.78
D-SAME (Figure 5)	1735	972	81.29	135.22

**Table 3:** Comparative analysis of synchronization and acceleration measurement results.

Work	Device	LUTs	FFs	$F_{\max}$ (MHz)	Power (mW)
[17]	Virtex-5 ^*a*^	50	47	–	–
[18]	ZYNQ-7000 ^*a*^	4468	4541	–	–
[19]	Spartan-3 ^*a*^	4777	2580	43.80	–
[20]	Artix-7 ^*a*^	2760	1305	39.95	75.00
This Work (Figure 3)	Cyclone V ^*b*^	930	522	119.70	130.81
This Work (Figure 4)	Cyclone V ^*b*^	1163	606	85.83	133.78

^*a*^ AMD Inc., San José, CA, USA. ^*b*^ Altera Inc., San José, CA, USA.

**Table 4:** Synchronization error under varying weather conditions.

	1PPS vs. Ext_1Hz (µs)				Ext_Sync_1 vs. Ext_Sync_2 (µs)				Ext_1Hz_1 vs. Ext_1Hz_2 (µs)			
Weather	Min/Max	Mean	Median	Std. Dev.	Min/Max	Mean	Median	Std. Dev.	Min/Max	Mean	Median	Std. Dev.
Cloudy	0.0/10.50	4.04	3.93	2.59	0.0/11.74	4.09	3.88	2.61	0.0/8.40	3.28	2.98	2.32
Moderate rain	0.0/11.46	4.28	4.14	2.49	0.0/13.28	5.18	4.70	3.40	0.0/14.18	6.14	5.88	3.30
Clear sky	0.0/10.48	3.89	3.69	2.35	0.0/6.98	2.30	2.04	1.70	0.0/7.14	2.21	1.92	1.65

**Table 5:** One-way ANOVA results for synchronization error analysis.

Setup	*F*-Value	*p*-Value
1PPS vs. Ext_1Hz	F(2,2685)=5.49	0.0048
Ext_Sync_1 vs. Ext_Sync_2	F(2,2688)=267.56	<0.0010
Ext_1Hz_1 vs. Ext_1Hz_2	F(2,2547)=553.23	<0.0010

**Table 6:** Measured frequency deviation of the generated timing signals.

Signal	Nominal Frequency	Mean Measured Frequency	Maximum Measured Frequency	Minimum Measured Frequency	Relative Deviation (ppm)
Ext_Clk	1.024000 MHz	1.024485 MHz	1.046666 MHz	1.020408 MHz	473.63
Ext_Sync	1.000000 kHz	1.000010 kHz	1.000020 kHz	1.000000 kHz	10.00
Ext_1Hz	1.000000 Hz	1.000006 Hz	1.000032 Hz	0.999980 Hz	5.99

**Table 7:** Mean acceleration and standard deviation for the +Z sensor orientation.

Test No.	Std. Dev. *X*-Axis	Std. Dev. *Y*-Axis	Std. Dev. *Z*-Axis	Mean *X*-Axis	Mean *Y*-Axis	Mean *Z*-Axis
1	2.3447	1.6690	3.1251	15.0515	−33.8569	995.5329
2	2.2324	1.6268	3.0663	15.0515	−33.8673	995.5479
3	2.1872	1.6063	3.0718	15.0368	−33.8721	995.5127
4	2.2115	1.6116	3.0469	15.0119	−33.8889	995.5163
5	2.2317	1.5864	2.9319	15.0319	−33.8869	995.1411

All values are expressed in mg.

## Data Availability

The data presented in this study are available on request from the corresponding author.
